# Exploring the metaverse in the education of healthcare students: A scoping review ^*^

**DOI:** 10.1590/1518-8345.7256.4347

**Published:** 2024-10-25

**Authors:** Andrea Bernardes, Lucas Gardim, Agostinho A C Araújo, Rodrigo Jensen, Raquel Acciarito Motta, Denise Maria de Almeida, Roberta Rubia de Lima, Heloísa Helena Ciqueto Peres

**Affiliations:** ^1^ Universidade de São Paulo, Escola de Enfermagem de Ribeirão Preto, PAHO/WHO Collaborating Centre for Nursing Research Development, Ribeirão Preto, SP, Brazil.; ^2^ Scholarship holder at the Conselho Nacional de Desenvolvimento Científico e Tecnológico (CNPq), Brazil.; ^3^ Scholarship holder at the University of Alberta Research Award Recipient (NOC 41201), Emerging Leaders in the Americas Program (ELAP) Program, Global Affairs Canada’s International Scholarships Program, Canada.; ^4^ Universidade de São Paulo, Escola de Enfermagem, São Paulo, SP, Brazil.; ^5^ Prisma Consultoria, São Paulo, SP, Brazil.; ^6^ Secretaria de Saúde do Estado de São Paulo, São Paulo, SP, Brazil.

**Keywords:** Education, Teaching, Students, Health Occupations, Technology, Educational Technologies, Virtual Reality

## Abstract

**Objective:**

to map the literature on the incorporation of the metaverse in the education of undergraduate healthcare students.

**Method:**

scoping review following the recommendations of the JBI and Preferred Reporting Items for Systematic Reviews and Meta-Analyses Extension for Scoping Reviews (PRISMA-ScR), performed on Web of Science, Medical Literature Analysis and Retrieval System Online (MEDLINE) via PubMed, Embase, Scopus, Cumulative Index to Nursing and Allied Health (CINAHL), Latin American and Caribbean Health Sciences Literature (LILACS) and ProQuest.

**Results:**

a total of 23 records were included, published between 2020 and 2023, and developed in 10 countries. The metaverse allows the simulation of hypothetical cases, making education interactive and attractive. However, it faces limitations, including the possibility of depersonalizing students, concerns about data security and privacy, and the high cost of implementing and maintaining its infrastructure.

**Conclusion:**

the metaverse enables the development of clinical competencies that support the construction of students’ professional identity. However, it may not be equitable, as it requires resources and knowledge from educators to implement it, contributing to increasing inequality in the education of healthcare students.

## Highlights

**(1)** The metaverse is promising in the education of undergraduate healthcare students.

**(2)** The metaverse makes education interactive and attractive.

**(3)** It promotes the protagonism of students in the teaching-learning process.

**(4)** It presents risks related to data security and privacy.

**(5)** High cost of implementing and maintaining its infrastructure.

## Introduction

 The world is constantly evolving technologically, which has not only influenced the way people relate to each other but also the teaching-learning process, especially in the education of undergraduate healthcare students ^(^ ^1^ ^-^ ^2^ ^)^ . The metaverse is one of the most recent and promising innovations, offering various possibilities and resources beyond physical and geographical restrictions ^(^ ^3^ ^)^ . For this reason, as this is an innovative topic, even though students belong to a generation with greater technological preparation, it is essential to have a scientific understanding of the use of this environment in education, given the scientific evidence with positive outcomes related to the use of educational technologies in improving the quality of care and the quality of life of patients ^(^ ^4^ ^)^ . 

 The metaverse is a technological educational environment composed of virtual and real forms, with almost indistinguishable boundaries ^(^ ^3^ ^)^ . Although seen as an emerging theme, the term “metaverse” was introduced in 1992 in a well-known work of science fiction by Neal Stephenson ^(^ ^5^ ^)^ . The metaverse is reiterated as a technological educational environment with the potential to revolutionize the teaching-learning process, creating immersive and interactive virtual environments that promote student motivation and engagement, thus favoring personalized learning ^(^ ^6^ ^)^ . 

 As a three-dimensional, interactive, and immersive technological environment, the metaverse offers a range of possibilities in which people can connect, collaborate, and experience engaging educational experiences ^(^ ^7^ ^-^ ^9^ ^)^ . In the context of training healthcare professionals, evidence demonstrates the effectiveness of this reality in proposing the teaching-learning process, as it enables the transition from theory to practice ^(^ ^10^ ^-^ ^11^ ^)^ . Nevertheless, some ethical repercussions relating to the metaverse application have been mentioned in various areas of knowledge, as well as limitations related to the scarcity of resources for its implementation and sustainability ^(^ ^8^ ^,^ ^12^ ^)^ . 

 Although review studies have been developed showing the application and use of the metaverse as an educational environment ^(^ ^13^ ^-^ ^14^ ^)^ , they do not synthesize the evidence in the context of the education of undergraduate healthcare students. These studies have shown that, due to its ability to facilitate innovative social interactions and provide immersive experiences ^(^ ^13^ ^)^ , the metaverse has been adapted for use in the context of education over time ^(^ ^14^ ^)^ . For this reason, this study aimed to map the literature on the incorporation of the metaverse into the education of undergraduate healthcare students. 

## Method

 This scoping review was conducted in compliance with the recommendations of the JBI ^(^ ^15^ ^)^ and reported in accordance with the guidelines of the Preferred Reporting Items for Systematic Reviews and Meta-Analyses Extension for Scoping Reviews (PRISMA-ScR) ^(^ ^16^ ^)^ . 

### Protocol

 Preliminary searches were carried out in the literature and no protocol or study relating to the incorporation of the metaverse in the education of undergraduate healthcare students was identified. The protocol for this study was then registered in the Open Science Framework (OSF) ^(^ ^17^ ^)^ , as per the recommendations of the JBI ^(^ ^15^ ^)^ . 

### Research question

 The acronym PCC (Population, Concept, and Context) ^(^ ^15^ ^)^ was used to establish the research question, whose “P” refers to undergraduate students; “C” to the metaverse; and “C” to healthcare education. 

 The higher education courses included in the healthcare context were defined based on the classification proposed by the World Health Organization (WHO) in 2019 ^(^ ^18^ ^)^ , which aims to map the occupations of health workers based on the differences assumed in the level of skills required for professional performance on a global scale. Thus, the following were considered as health courses: nursing, pharmacy, physical therapy, speech therapy, medicine (alternative and generalist), midwifery, nutrition, dentistry, and occupational therapy. 

 The definition of metaverse followed the concept described by Zhang X, Chen Y, Hu L, and Wang Y ^(^ ^3^ ^)^ , which highlights such reality as an educational environment composed by up-to-date technologies, which are essential for ensuring components in the real and fictional world with a view to a successful educational experience. Therefore, given the scientific progress and consequent continuous implementation of digital strategies in the context of education, there is a need to understand not only the applicability of the metaverse but also its limitations so that it can be incorporated into the education of undergraduate healthcare students. 

Thus, the following research question was established: “What is the state of knowledge regarding the incorporation of the metaverse in the education of undergraduate healthcare students?”. This question was subdivided into sub-questions:

What is the applicability of the metaverse in the education of undergraduate healthcare students?

What are the limitations of the metaverse in the education of undergraduate healthcare students?

## Search strategy

A preliminary search was conducted on the Medical Literature Analysis and Retrieval System Online (MEDLINE) via PubMed in July 2023 to identify the usual descriptors adopted by the scientific community related to the topic, considering that, at the time of the literature searches, “metaverse” was not a descriptor. Meetings were held between the research team and an experienced librarian to establish the search strategy, which was adjusted and standardized according to the different databases consulted.

The searches were carried out on July 20, 2023, in ISI Web of Science, MEDLINE via PubMed, Embase, Scopus, EBSCOhost Cumulative Index to Nursing and Allied Health (CINAHL), BVShost Latin American and Caribbean Health Sciences Literature (LILACS), and ProQuest. The search terms included: “metaverse*, metaverse environment, metaverse experience, metaverse for learning, metaverse healthcare, metaverse in education, metaverse technolog*”, “educa*, workshop*, training program*, educational activit*, literacy program*, education service, intellectual training, training support.”, and “health”. Descriptors and/or keywords were combined according to the Medical Subject Headings (MeSH), CINAHL Titles, Emtree, and Health Sciences Descriptors (DeCS) index terms available in each database.

### Selection criteria

Records that mapped the applicability and limitations of the metaverse in the education of undergraduate healthcare students published in Portuguese, English, and/or Spanish (the authors’ languages of proficiency) were included. Records relating strictly to Virtual Reality (VR) and Augmented Reality (AR) for the education of undergraduate healthcare students were excluded. It should be noted that no time frame was adopted.

### Selection of evidence sources

 The identified records were exported to EndNote ^®^ Web to remove duplicates. Subsequently, the files were transferred to Rayyan ^(^ ^19^ ^)^ for simultaneous and independent screening by two reviewers, to improve reliability and methodological accuracy. A third reviewer with expertise in the subject was consulted to resolve disagreements in the sample definition process. The screening process for the full texts followed the same procedures. A hand search was performed, and the remaining records composed the final sample. 

### Data extraction and analysis

The records included were analyzed and their data was extracted by one reviewer and validated by a second, considering: (1) characteristics of the record: author, year of publication, and country (origin of the first author); (2) publication: type (discussion or research study), design, population (characterization and number of participants, if applicable), and main findings; (3) applicability of the metaverse in the education of undergraduate healthcare students; and (4) limitations of the metaverse in the education of undergraduate healthcare students. The data was analyzed using descriptive and content analysis, which included information on the applicability and limitations of the metaverse in the education of undergraduate healthcare students.

## Results

**Figure 1:**
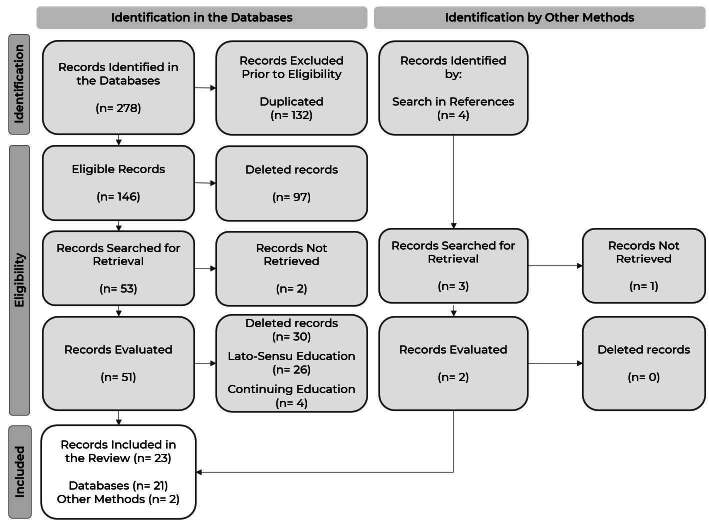
- Preferred Reporting Items for Systematic Reviews and Meta-Analyses Extension for Scoping Reviews (PRISMA-ScR) ^(^ ^16^ ^)^ . Ribeirão Preto, SP, Brazil, 2023

 The results of this study were guided by PRISMA-ScR ^(^ ^16^ ^)^ , whose review process is described in Figure 1 . 

 Of the 278 records mapped, 23 were included in this review ^(^ ^20^ ^-^ ^42^ ^)^ . These were published between 2020 and 2023 in 10 countries, spread across four continents: Asia (11, ~48%) ^(^ ^23^ ^-^ ^26^ ^,^ ^39^ ^,^ ^42^ ^)^ , Europe (5, ~22%) ^(^ ^27^ ^-^ ^29^ ^,^ ^40^ ^-^ ^41^ ^)^ , America (4, ~17%) ^(^ ^30^ ^-^ ^33^ ^)^ , and Oceania (3, ~13%) ^(^ ^20^ ^-^ ^22^ ^)^ . Most of the records included were scientific studies (17, ~74%) ^(^ ^23^ ^-^ ^29^ ^,^ ^33^ ^-^ ^42^ ^)^ . Among the methods used, literature review studies were prevalent (16, ~70%) ^(^ ^23^ ^,^ ^25^ ^,^ ^27^ ^-^ ^28^ ^,^ ^33^ ^-^ ^42^ ^)^ . Although the type of review was not characterized by the authors for the most part (10, ~44%) ^(^ ^23^ ^,^ ^25^ ^,^ ^28^ ^,^ ^34^ ^-^ ^38^ ^,^ ^41^ ^-^ ^42^ ^)^ , scoping (2, ~9%) ^(^ ^39^ ^-^ ^40^ ^)^ , systematic (1, ~4%) ^(^ ^27^ ^)^ , and umbrella reviews (1, ~4%) ^(^ ^33^ ^)^ composed the final sample. 

 Considering that this review included several healthcare courses in a multidisciplinary context, it can be seen that, for most of the productions (18, ~78%) ^(^ ^20^ ^-^ ^21^ ^,^ ^23^ ^,^ ^25^ ^,^ ^27^ ^-^ ^28^ ^,^ ^30^ ^-^ ^33^ ^,^ ^35^ ^-^ ^42^ ^)^ , the definition of the population by course does not apply, considering that literature review studies were prevalent. However, a descriptive analysis shows that the medical course is predominant (14, ~61%) ^(^ ^21^ ^,^ ^25^ ^,^ ^28^ ^-^ ^32^ ^,^ ^34^ ^-^ ^36^ ^,^ ^38^ ^-^ ^41^ ^)^ compared to nursing (3, ~13%) ^(^ ^20^ ^,^ ^24^ ^,^ ^33^ ^)^ , and dentistry (1, ~4%) ^(^ ^42^ ^)^ . Figure 2 shows the characteristics of the records included in this review. 

**Figure 2:** - Characteristics of the records included. Ribeirão Preto, SP, Brazil, 2023

**Authors, year of publication, and country**	**Type of publication**	**Design**	**Population**	**Main findings**
van Weelderen, 2022 ^(^ ^20^ ^)^ Australia	Discussion	Opinion article	Not applicable	It points out that, with the progress of technology, education has advanced and, as a result, the role of nursing has changed exponentially.
Mah, 2023 ^(^ ^21^ ^)^ Australia	Discussion	Editorial	Not applicable	It highlights that the integration of Artificial Intelligence (AI) into education, training, and research is expected to revolutionize medical education and practice, providing better outcomes for patients.
Moro, 2023 ^(^ ^22^ ^)^ Australia	Discussion	Opinion article	Not detailed	It demonstrates that the metaverse can offer opportunities for teaching and learning anatomy and physiology, although there are some risks to consider.
Chen, Zou, Xie, Wang, 2023 ^(^ ^23^ ^)^ China	Research study	Review study	Not applicable	It evidences that the metaverse promotes collaborative and playful experiences, as well as the development of skills, although challenges need to be resolved. The findings highlight the need for safety measures and technological training for instructors, in addition to exploring the metaverse in monitoring and analyzing student behavior.
Yang, Kang, 2022 ^(^ ^24^ ^)^ South Korea	Research study	Quasi-Experimental	58 third-year nursing students who completed theoretical classes on schizophrenia and mental health related to therapeutic communication, 29 students for the experimental group, and 29 students for the control group.	It shows that the metaverse presents positive results that indicate its usefulness as an alternative in nursing education, overcoming the space-time limitations of clinical practice. However, it also shows that current technological limitations affect the naturalness of interactions.
Musamih, Yaqoob, Salah, Jayaraman, Al-Hammadi, Omar, et al., 2023 ^(^ ^25^ ^)^ United Arab Emirates	Research study	Review study	Not applicable	It reiterates the likely application of the metaverse in various healthcare fields, such as telemedicine, medical education, healthcare marketing, supply chain, healthcare facilities, and wellness. However, it shows that widespread adoption faces significant challenges.
Al-Kfairy, Al-Fandi, Alema, Altaee, 2023 ^(^ ^26^ ^)^ United Arab Emirates	Research study	Qualitative study	84 higher education students. However, there were no details of the respective courses to which they were linked.	It points out that learning flexibility is the main incentive for using metaverse-based classrooms, with other motivations including concern for the environment and the interactivity offered by this form of technology. However, concerns about possible health problems are highlighted, as well as privacy and security issues.
Gonzalez-Moreno, Andrade-Pino, Monfort-Vinuesa, Piñas-Mesa, Rincon, 2023 ^(^ ^27^ ^)^ Spain	Research study	Systematic review	Not applicable	It emphasizes that to implement successful metaverse-related strategies in education, it is necessary to consider the various mixed reality techniques that have been developed, addressing their potential and weaknesses.
Román-Belmonte, Rodríguez-Merchán, Corte-Rodríguez, 2023 ^(^ ^28^ ^)^ Spain	Research study	Review study	Not applicable	It points to the use of the metaverse in musculoskeletal pathology education as promising for improving knowledge acquisition and promoting independent learning. It also highlights clinical applications under development that could add significant value to the field of osteoarticular medicine. Furthermore, it highlights the use of the metaverse as promising, despite clinical, technological, educational, and legal issues that still need to be addressed.
Méndez, Marcos-Pablos, Izard, 2023 ^(^ ^29^ ^)^ Spain	Research study	Proceedings of the 2022 Tenth International Conference on Technological Ecosystems for Enhancing Multiculturality (TEEM-2022) Survey	Not detailed	It points out that the metaverse is effective in enhancing the visualization of real images in virtual learning environments. The study presents several immersive and augmented virtual environments, using stereoscopic models to create a metaverse where knowledge can be shared to improve medical training.
López-Ojeda, Hurley, 2023 ^(^ ^30^ ^)^ United States of America (USA)	Discussion	Commentary	Not applicable	It points out that three-dimensional printing technology, combined with AI, is developing rapidly in the context of the metaverse, driving advances in medical education and health care. Although it cannot yet completely replace the use of human cadavers in medical training, the study highlights the metaverse as an innovative complement to disciplines such as clinical neuroscience.
Ford, Buchanan, Azeez, Benrimoh, Kaloiani, Bandeira, et al., 2023 ^(^ ^31^ ^)^ United States of America (USA)	Discussion	Perspective	Not applicable	It emphasizes that the metaverse has emerged on the medical education scene as an educational environment that allows students to experience situations that are close to reality, corroborating their clinical reasoning.
Ahuja, Polascik, Doddapaneni, Byrnes, Sridhar, 2022 ^(^ ^32^ ^)^ United States of America (USA)	Discussion	Commentary	Not applicable	It points out that the metaverse contributes to revolutionizing medical education when combined with AI, AR, and VR. It also points out that, although there is still room for growth and consideration of disadvantages, its adoption can expand access to medical education for underserved populations.
De Gagne, Randall, Rushton, Park, Cho, Yamane, et al., 2022 ^(^ ^33^ ^)^ United States of America (USA)	Research study	Umbrella review	Not applicable	It highlights the metaverse as a versatile and constantly evolving pedagogical tool with the potential to boost learning outcomes in nursing education, opening up fascinating opportunities and important considerations for educators.
Bhatia, Joshi, 2023 ^(^ ^34^ ^)^ India	Research study	Anais do International Conference on Innovation Data Communication Technologies and Application 2023 (ICIDCA-2023) Review study	Not detailed	It highlights that medical students’ practice in the metaverse transforms medical training, saving time and resources.
Kawarase, Anjankar, 2022 ^(^ ^35^ ^)^ India	Research study	Review study	Not applicable	It highlights that considering cultural differences in the global context, the metaverse can offer an educational environment that provides quality and uniformity in medical education.
Pandey, Chirputkar, Ashok, 2023 ^(^ ^36^ ^)^ India	Research study	Review study	Not applicable	It emphasizes that the metaverse corresponds to an educational approach that will influence medical education in the future, even if it is related to improving some personal characteristics.
Mohamed, Naqishbandi, Veronese, 2023 ^(^ ^37^ ^)^ India	Research study	Review study	Not applicable	It highlights the metaverse as a new and exceptional environment for education. It also shows that the topic is increasingly developing, especially in terms of applications, the importance of which has increased significantly due to the COVID-19 pandemic.
Bhugaonkar, Bhugaonkar, Masne, 2022 ^(^ ^38^ ^)^ India	Research study	Review study	Not applicable	It points out that the metaverse is intrinsically associated with AR and can therefore be applied to the educational process of medical students.
Garavand, Aslani, 2022 ^(^ ^39^ ^)^ Iran	Research study	Scope review	Not applicable	It highlights that, in medical education, the use of the metaverse can revolutionize the way students learn and practice medicine, benefiting public health.
Petrigna, Musumeci, 2022 ^(^ ^40^ ^)^ Italy	Research study	Scope review	Not applicable	It emphasizes that the metaverse reduces the discrepancy in medical training, considering the elimination of geographical and time barriers through virtual learning rooms.
Massetti, Chiariello, 2023 ^(^ ^41^ ^)^ Italy	Research study	Review study	Not applicable	It points out that, in education, the metaverse enables students to experience clinical situations safely, gaining experience in the teaching-learning process.
Bansal, Rajgopal, Chamola, Xiong, Niyato, 2020 ^(^ ^42^ ^)^ Singapore	Research study	Review study	Not applicable	It shows that the metaverse has emerged as an alternative for developing and improving existing educational systems, enabling an experience that resembles reality.

Figure 3 shows the summary table of the records included in the final sample. The results elucidate the applicability and limitations of the metaverse in the education of undergraduate healthcare students. Although some studies detail only one of the axes observed, the majority (15, ~65%) ^(^ ^21^ ^-^ ^25^ ^,^ ^29^ ^,^ ^31^ ^-^ ^32^ ^,^ ^34^ ^-^ ^35^ ^,^ ^37^ ^-^ ^40^ ^,^ ^42^ ^)^ describe both applicability and limitations in their entirety. 

**Figure 3:** - Applications and limitations of the metaverse in the education of undergraduate healthcare students. Ribeirão Preto, SP, Brazil, 2023

**Authors and year of publication**	**Applicability of the metaverse**	**Limitations of the metaverse**
van Weelderen, 2022 ^(^ ^20^ ^)^	Development of skills through the execution of clinical procedures; Reduces the risk of injury to students by providing a safe environment for practicing and learning to make critical decisions before interacting with real patients; Reduces stress and improves staff retention rates.	Not described
Mah, 2023 ^(^ ^21^ ^)^	Simulates real-life surgical scenarios in medical education; Allows repeated practice with unlimited resources.	It can confuse students’ understanding of the difference between the metaverse and real life. Encourages academic fraud through cheating on exams.
Moro, 2023 ^(^ ^22^ ^)^	Virtual dissections; Consultation with expert instructors from anywhere in the world; Understanding anatomy through the representation of 3D organs from different anatomical models.	Cost of implementation; Demand for stable connectivity; Data protection, privacy and security; Difficulty in monitoring students in the virtual world.
Chen, Zou, Xie, Wang, 2023 ^(^ ^23^ ^)^	Enables the expansion of students’ clinical skills, based on real-life situations.	Difficulties in security and privacy protection; May confuse students’ understanding of what is real and virtual.
Yang, Kang, 2022 ^(^ ^24^ ^)^	Enables students to improve their therapeutic communication with psychiatric patients.	Facial expressions and body postures of the avatars are limited and far from natural compared to the patients; Exposure of the programs to problems that may occur in the operational process during use.
Musamih, Yaqoob, Salah, Jayaraman, Al-Hammadi, Omar, et al., 2023 ^(^ ^25^ ^)^	Enables surgical training; Facilitates collaboration between educators and students from different locations.	Requires advanced and expensive technologies; Requires resources relating to the interoperability and compatibility of systems; Exposes to the risk of cybercrime (identity theft and data breaches, cyberbullying and harassment); Exposes the risk of virtual addiction.
Al-Kfairy, Al-Fandi, Alema, Altaee, 2023 ^(^ ^26^ ^)^	Not described	Difficulty in accessing and handling the technology; Usability problems ^*^ ; Data security and privacy.
Gonzalez-Moreno, Andrade-Pino, Monfort-Vinuesa, Piñas-Mesa, Rincon, 2023 ^(^ ^27^ ^)^	Enables the reproduction of Health Digital Twins (HDT) ^†^ .	Not described
Román-Belmonte, Rodríguez-Merchán, Corte-Rodríguez, 2023 ^(^ ^28^ ^)^	Provides training in orthopedic surgical skills by recognizing anatomical structures and performing fluoroscopic techniques and surgical sutures, as well as access to various surgical environments via a 360º camera; Provides training for students in situations considered rare, such as multiple victim simulations; Reduces the risk of accidental injuries with needles, scalpels or other sharp instruments, improving safety in invasive procedures.	Not described
Méndez, Marcos-Pablos, Izard, 2023 ^(^ ^29^ ^)^	Enables spatial visualization of real environments in the virtual world (e.g. operating rooms, anatomical dissection rooms, clinical examination rooms); Enables learning about the structures of the human body, such as the skeletal system and body musculature; Allows health science students to obtain information complementary to the regular content of their studies, becoming active participants in the learning process, and encouraging them during their educational progress.	Technical aspects still need to be solved to make the user’s subjective feeling of being physically present a reality.
López-Ojeda, Hurley, 2023 ^(^ ^30^ ^)^	Supports the training of surgical procedures in neurosurgery; Enables the printing of 3D anatomical models; Enables enhanced presentation of complex anatomical regions, organs, and structures.	Not described
Ford, Buchanan, Azeez, Benrimoh, Kaloiani, Bandeira, et al., 2023 ^(^ ^31^ ^)^	Promotes performance feedback; Promotes specialized teaching (neurosimulation); Promotes simulated teaching (collecting a previous history; carrying out a physical examination; generating diagnostic hypotheses and proposing therapeutic management for various diseases, including those considered rare); Promotes the improvement of clinical knowledge and ensures patient safety through learning opportunities.	Ethical implications related to accessibility and equity.
Ahuja, Polascik, Doddapaneni, Byrnes, Sridhar, 2022 ^(^ ^32^ ^)^	Enables the teaching of anatomy and radiology; Provides opportunities to improve clinical skills; Enables surgical training.	High implementation costs; Data security vulnerabilities.
De Gagne, Randall, Rushton, Park, Cho, Yamane, et al., 2022 ^(^ ^33^ ^)^	Not described	Risks of technology malfunction.
Bhatia, Joshi, 2023 ^(^ ^34^ ^)^	Enables surgical training (handling equipment, complex manual skills, practice on simulated patients with real patient data); Provides opportunities for remote communication and interaction with other students and patients; Simulation of real-life cases in the area of neurosurgical emergencies; Opportunities to connect people from different regions.	High cost of implementation; Risk to patient data privacy.
Kawarase, Anjankar, 2022 ^(^ ^35^ ^)^	Enables the simulation of scenarios and case conditions that resemble real life; Enables training in surgical procedures; Enables the teaching of anatomy of specific structures, such as lymph nodes, blood vessels, nerves, microscopic structures, and pathologies.	Costly to implement (infrastructure and personnel); Risk of security and data privacy breaches.
Pandey, Chirputkar, Ashok, 2023 ^(^ ^36^ ^)^	Supports the teaching of human anatomy; Simulates surgical procedures.	Not described
Mohamed, Naqishbandi, Veronese, 2023 ^(^ ^37^ ^)^	Provides an assimilation between the real and virtual worlds.	Complex and not fully understood technologies with the potential to profoundly influence life.
Bhugaonkar, Bhugaonkar, Masne, 2022 ^(^ ^38^ ^)^	Enables visualization of the anatomical structure of the human body; Enables training in surgical techniques; Makes it possible to simulate high-cost surgical procedures.	Difficulty or impossibility of exporting content between metaverses; Risks related to the privacy and protection of patient data; High cost of implementation; Environment vulnerable to cyber-attacks.
Garavand, Aslani, 2022 ^(^ ^39^ ^)^	Provided opportunities for non-face-to-face education in the context of the COVID-19 pandemic.	Need for infrastructure to apply the metaverse in different areas.
Petrigna, Musumeci, 2022 ^(^ ^40^ ^)^	Enables training in cardiology and neurology surgeries; Enables visualization of anatomical structures of the human body.	Weakens social connections compared to real life.
Massetti, Chiariello, 2023 ^(^ ^41^ ^)^	Enables interactive digital anatomy classes, to understand the evolution of the pathological process and simulate procedures; Simulates clinical and diagnostic activities on virtual models using virtual patients with similar characteristics to real patients.	Not described
Bansal, Rajgopal, Chamola, Xiong, Niyato, 2020 ^(^ ^42^ ^)^	Simulated cases with screen projections that allow monitoring of what students are doing; Teaching about the positioning of the placenta and the stages of childbirth; Dental simulators that respond to the force applied by the student when simulating the procedure and allow them to train procedures and dexterity; Teaching about emergency care such as cardiopulmonary resuscitation, use of a defibrillator, collecting tests, studying anatomy and dissection, surgical training.	Issues relating to privacy and data security, laws, and jurisdiction; Risks related to addiction and mental health.

^*^ Usability = Refers to the ease of using new technology ^(^ ^26^ ^)^ ;

^†^ Health Digital Twins (HDT) = A virtual representation (digital twin) of a patient (physical twin) that is generated from multimodal patients ^(^ ^27^ ^)^

## Discussion

The results of this study show that only three of the nine courses included in the classification of higher education courses in the healthcare area have incorporated the metaverse into their students’ education; a scenario in which medicine takes the lead. It can therefore be seen that the incorporation of the metaverse in education is incipient and, as a result, investments are needed to ensure that education in undergraduate healthcare courses is equitable. The evidence summarized highlights the applicability and limitations of the metaverse in higher education in the healthcare context, showing a reality that permeates theory and practice.

## Applicability of the metaverse in the education of undergraduate healthcare students

 Indeed, the metaverse supports education by simulating hypothetical cases that resemble reality ^(^ ^21^ ^,^ ^23^ ^,^ ^27^ ^-^ ^28^ ^,^ ^34^ ^-^ ^35^ ^,^ ^37^ ^,^ ^41^ ^)^ , thus contributing to the development of students’ competencies. Among the main applications of the metaverse, anatomy teaching ^(^ ^22^ ^,^ ^28^ ^-^ ^30^ ^,^ ^32^ ^,^ ^35^ ^-^ ^36^ ^,^ ^38^ ^,^ ^40^ ^-^ ^42^ ^)^ was largely explored by the records included, given its ability to facilitate understanding of the dimensions of the human body through anatomical visualization at different angles. This strategy is an advance on traditional teaching with cadavers, especially as it makes it possible to observe microscopic structures ^(^ ^22^ ^,^ ^43^ ^)^ . 

Still from a theoretical perspective, the metaverse contributes to making the teaching-learning process not only interactive but also attractive. Thus, students’ intuition and imagination must be awakened and stimulated so that they can be motivated to learn since the metaverse has a lot to contribute to academic performance as a technological educational environment. However, for that to happen, students must accept the metaverse.

 Although the applicability of the metaverse is related to theoretical teaching, it enhances the development of clinical competencies ^(^ ^20^ ^,^ ^23^ ^-^ ^24^ ^,^ ^28^ ^-^ ^32^ ^,^ ^34^ ^-^ ^35^ ^,^ ^38^ ^,^ ^40^ ^,^ ^42^ ^)^ , such as surgical training (regardless of the specialty) ^(^ ^25^ ^,^ ^28^ ^-^ ^32^ ^,^ ^34^ ^-^ ^36^ ^,^ ^38^ ^,^ ^40^ ^,^ ^42^ ^)^ and training in emergencies with multiple victims ^(^ ^31^ ^)^ . By allowing procedures to be repeated ^(^ ^21^ ^)^ without causing any risk to the patient or student ^(^ ^20^ ^,^ ^28^ ^,^ ^31^ ^)^ , the metaverse allows the dynamics of the clinical scenario to be monitored, making it possible to understand the procedures and the size of the work team and even contributing to teaching patient safety. 

 Established in 2011 by the World Health Organization (WHO), the Multi-Professional Patient Safety Curriculum Guide emphasizes that patient safety should be incorporated into the pedagogical proposals of interprofessional health education ^(^ ^44^ ^)^ . This makes it possible to train professionals who can act in accordance with best practices, not only providing safe and qualified patient care but also contributing to strengthening health systems. For this reason, it is essential to invest in incorporating patient safety into the teaching-learning process ^(^ ^45^ ^-^ ^47^ ^)^ , with the metaverse being a strategy. 

 In the context of the COVID-19 pandemic, the imminent need for non-face-to-face education has led to reflections on the teaching-learning process ^(^ ^39^ ^)^ . The metaverse has been boosted by the dizzying search for ways to facilitate interaction and collaboration between educators and students in different locations ^(^ ^25^ ^)^ . As a result, the boundaries between the real and virtual worlds have sometimes become almost indistinguishable, increasing education and networking ^(^ ^34^ ^)^ between students and educators around the world through emergency remote teaching ^(^ ^22^ ^)^ . 

 The synthesis of evidence covered three of the four possible applications of the metaverse: blended learning, competency-based education, and inclusive education ^(^ ^3^ ^)^ . In terms of theoretical applicability, the metaverse can be a technological educational environment aimed at promoting student protagonism in the teaching-learning process. In addition, concerning practical applicability, it enables the development of clinical competencies that support the construction of professional identity, reiterating its commitment to professionalism. 

### Limitations of the metaverse in the education of undergraduate healthcare students

 Certainly, there is still much progress to be made in incorporating the metaverse into the education of undergraduate healthcare students. Therefore, the idea of only emphasizing infrastructure as a necessary factor for the metaverse to be used in education must be disassociated. Antagonistically, educators must have the skills to use this technological educational environment as a pedagogical resource, especially when considering the possibility of weakening interpersonal relationships in the educator-student dyad. For this reason, educators need to develop basic competencies that must be imbued with pedagogical knowledge ^(^ ^48^ ^)^ . 

 Although the Multi-Professional Patient Safety Curriculum Guide does not specifically mention the metaverse, this document highlights the difficulty students have in discerning between virtual and physical reality as a concern that should be considered by educators ^(^ ^44^ ^)^ . The use of the metaverse by educators who do not have specific knowledge can lead to a process of depersonalization of students ^(^ ^21^ ^-^ ^23^ ^,^ ^32^ ^,^ ^40^ ^)^ . As a result, students can be detached from reality by feeling disconnected from their own bodies and thoughts, since the environment and activities inherent in the metaverse “resemble” real life (even if it has limitations) ^(^ ^24^ ^,^ ^29^ ^,^ ^33^ ^,^ ^37^ ^)^ , making it difficult to equate what is real with what is virtual. 

 Nevertheless, when it comes to the limitations of the metaverse in the education of undergraduate healthcare students, data security and privacy ^(^ ^22^ ^-^ ^23^ ^,^ ^25^ ^,^ ^31^ ^-^ ^32^ ^,^ ^34^ ^-^ ^35^ ^,^ ^38^ ^,^ ^42^ ^)^ must be highlighted as a restrictive factor. As the real world is reproduced in the metaverse, risks related to cybercrime, such as identity theft and data breaches, as well as cyberbullying and harassment ^(^ ^25^ ^,^ ^38^ ^)^ , can be triggered. For this reason, maintenance and protection resources must be considered for the metaverse to be implemented. In the global context, higher education is not equal, as it is related to economic development ^(^ ^49^ ^)^ . In this sense, when considering the metaverse as a technological educational environment, limiting factors include not only the high cost of implementation ^(^ ^22^ ^,^ ^25^ ^,^ ^32^ ^,^ ^34^ ^-^ ^35^ ^,^ ^38^ ^-^ ^39^ ^)^ , but also the maintenance of the technology. 

Despite its applicability, the metaverse has limitations that must be considered. For its adoption to be effective, educators must be instructed in the use of technology to minimize negative repercussions for students. Implementation measures must be taken along with maintenance strategies so that data security and privacy can be guaranteed. Finally, the use of the metaverse may not be equitable, insofar as not all Higher Education Institutions (HEIs) have resources, conditions, and educators with the skills to implement it, which may contribute to accentuating inequality in the education of undergraduate healthcare students.

This study did not include analysis by courses in the healthcare context, as well as physical and psychological aspects related to the use of the metaverse (effects induced by the virtual environment: cybersickness, simulator sickness, movement sickness, cognitive loss, dizziness, physical discomfort). Furthermore, as this is an emerging topic in various areas of knowledge, the concept of “metaverse” is still fluid, as evidence is continually being developed to qualify it. This may have limited the search for records.

## Conclusion

The metaverse contributes to making the teaching-learning process for undergraduate healthcare students not only interactive but also attractive. This enables the development of clinical competencies that support the construction of professional identity. Despite this, the metaverse has limitations that need to be considered, especially data security and privacy and the high cost associated with its implementation and maintenance. The metaverse may not be equitable, as not all HEIs have the resources, conditions, and professionals with the skills to implement it, which may contribute to accentuating inequality in education. The different applications of the metaverse have proven promising for proposing qualified higher education in the global context. However, as an evolving field, there are still challenges in integrating it into the education of undergraduate healthcare students.
