# Supplementary material for: Exploring the metaverse in the education of healthcare students: A scoping review
Source: Rev Lat Am Enfermagem. 2024 Oct 25;32:e4347. doi: 10.1590/1518-8345.7256.4347 (PMC11526252; doi:10.1590/1518-8345.7256.4347)
Supplement: Translation

# Translation: Explorando el metaverso en la educación de estudiantes de salud: revisión de alcance ^*^

**DOI:** 10.1590/1518-8345.7256.4346

**Authors:** Andrea Bernardes, Lucas Gardim, Agostinho A C Araújo, Rodrigo Jensen, Raquel Acciarito Motta, Denise Maria de Almeida, Roberta Rubia de Lima, Heloísa Helena Ciqueto Peres

**Affiliations:** ^1^ Universidade de São Paulo, Escola de Enfermagem de Ribeirão Preto, Centro Colaborador de la OPS/OMS para el Desarrollo de la Investigación en Enfermería, Ribeirão Preto, SP, Brasil.; ^2^ Becaria del Conselho Nacional de Desenvolvimento Científico e Tecnológico (CNPq), Brasil.; ^3^ Becario de la University of Alberta Research Award Recipient (NOC 41201), Emerging Leaders in the Americas Program (ELAP) Program, Global Affairs Canada’s International Scholarships Program, Canadá.; ^4^ Universidade de São Paulo, Escola de Enfermagem, São Paulo, SP, Brasil.; ^5^ Prisma Consultoria, São Paulo, SP, Brasil.; ^6^ Secretaria de Saúde do Estado de São Paulo, São Paulo, SP, Brasil.

**Keywords:** Educación, Enseñanza, Estudiantes del Área de la Salud, Tecnología, Tecnología Educacional, Realidad Virtual

## Abstract

**Objetivo:**

mapear la literatura respecto de incorporar el metaverso a la educación de estudiantes de carreras del área de salud.

**Método:**

revisión de alcance, acorde recomendaciones del JBI y *Preferred Reporting Items for Systematic Reviews and Meta-Analyses Extension for Scoping Reviews* (PRISMA-ScR), realizada en *Web of Science* , *Medical Literature Analysis and Retrieval System Online* (MEDLINE) vía PubMed, Embase, Scopus, *Cumulative Index to Nursing and Allied Health* (CINAHL), *Latin American and Caribbean Health Sciences Literature* (LILACS) y ProQuest.

**Resultados:**

fueron incluidos 23 registros, publicados entre 2020 y 2023, desarrollados en 10 países. El metaverso destaca por permitir simular casos hipotéticos, haciendo que la educación resulte interactiva y atractiva. Sin embargo, enfrenta limitaciones, incluyendo la posible despersonalización de los estudiantes, preocupación por la seguridad y privacidad de los datos, además del elevado costo de implementación y mantenimiento de su infraestructura.

**Conclusión:**

el metaverso posibilita el desarrollo de competencias clínicas que facilitan la construcción de identidad profesional del estudiante. No obstante, puede no ser equitativo, considerando que demanda recursos y educadores con conocimientos para implementarlo, contribuyendo a acentuar desigualdades en la educación de estudiantes.

## Destacados

**(1)** El metaverso es promisorio en la educación de estudiantes de carreras del área de la salud.

**(2)** El metaverso hace que la educación se muestre interactiva y atractiva.

**(3)** Promueve el protagonismo de los estudiantes en el proceso enseñanza-aprendizaje.

**(4)** Demuestra riesgos relacionados con la seguridad y privacidad de los datos.

**(5)** Costos elevados de implementación y mantenimiento de su infraestructura.

## Introducción

 El mundo está en constante evolución tecnológica, lo cual no solo ha influido en el modo en que las personas se relacionan, sino también en el proceso de enseñanza-aprendizaje, particularmente en la educación de estudiantes de carreras del área de la salud ^(^ ^1^ ^-^ ^2^ ^)^ . El metaverso surge como una de las más recientes y promisorias innovaciones, ofreciendo una gama de posibilidades y recursos que trascienden restricciones físicas y geográficas ^(^ ^3^ ^)^ . Por ello, tratándose de un tema de carácter innovador y aunque los estudiantes pertenezcan a una generación con mejor preparación tecnológica, es imprescindible la comprensión científica respecto del uso de dicho ambiente en la educación, vistas las evidencias científicas con desenlace positivo relativas al uso de tecnologías educativas en la mejora de la calidad de atención y de la calidad de vida de los pacientes ^(^ ^4^ ^)^ . 

 El metaverso está considerado un ambiente educativo tecnológico, constituido real y virtualmente, con límites casi indistinguibles ^(^ ^3^ ^)^ . Aunque se lo considera asunto emergente, el término “metaverso” fue acuñado en 1992, en una reconocida obra de ciencia ficción del autor Neal Stephenson ^(^ ^5^ ^)^ . En el contexto de la educación, se replica el metaverso como ambiente educativo tecnológico con potencial para revolucionar el proceso enseñanza-aprendizaje, creando ambientes virtuales inmersivos e interactivos, promotores de motivación y compromiso de los estudiantes, favoreciéndose así un aprendizaje personalizado ^(^ ^6^ ^)^ . 

 Como ambiente tecnológico tridimensional, interactivo e inmersivo, el metaverso ofrece una gama de posibilidades en las que las personas pueden conectarse, colaborar y vivir experiencias educativas envolventes ^(^ ^7^ ^-^ ^9^ ^)^ . En el contexto de la formación de profesionales de salud, las evidencias demuestran la eficacia de esta realidad para proponer el proceso de enseñanza-aprendizaje, ya que posibilita la transición de la teoría a la práctica ^(^ ^10^ ^-^ ^11^ ^)^ . No obstante, se han referido algunas repercusiones éticas sobre la aplicación del metaverso en diversas áreas del conocimiento, así como limitaciones relacionadas a la escasez de recursos para su implementación y sostenibilidad ^(^ ^8^ ^,^ ^12^ ^)^ . 

 En dicho sentido, si bien se han realizado estudios de revisión de literatura que muestran la aplicación y uso del metaverso como ambiente educativo ^(^ ^13^ ^-^ ^14^ ^)^ , no sintetizan las evidencias en el contexto de la educación de estudiantes de carreras del área de la salud. Dichos estudios demostraron que, debido a su capacidad para facilitar interacciones sociales innovadoras y brindar experiencias inmersivas ^(^ ^13^ ^)^ , el metaverso fue adaptado para su uso en el contexto de la educación a lo largo del tiempo ^(^ ^14^ ^)^ . Por tal razón, el objetivo de este estudio fue mapear la literatura sobre la incorporación del metaverso en la educación de estudiantes de carreras del área de la salud. 

## Método

 Esta revisión de alcance fue realizada conforme las recomendaciones del JBI ^(^ ^15^ ^)^ y presentada de acuerdo con las directrices del *Preferred Reporting Items for Systematic Reviews and Meta-Analyses Extension for Scoping Reviews* (PRISMA-ScR) ^(^ ^16^ ^)^ . 

### Protocolo

 Fueron realizadas búsquedas preliminares en la literatura y no fue identificado ningún protocolo o estudio relativo a la incorporación del metaverso en la educación de estudiantes de carreras del área de la salud. Seguidamente se procedió a registrar el protocolo de este estudio en el *Open Science Framework* (OSF) ^(^ ^17^ ^)^ atendiendo las recomendaciones del JBI ^(^ ^15^ ^)^ . 

### Pregunta de investigación

 Fue utilizado el acrónimo PCC (Población, Concepto y Contexto) ^(^ ^15^ ^)^ para establecer la pregunta orientadora, de forma que “P” se refiere a los educación de estudiantes de pregrado; “C” al metaverso; y “C” a la educación en el área de salud. 

 Vale destacar que las carreras de educación superior incluidas en el área de salud fueron definidas a partir de la clasificación propuesta por la Organización Mundial de la Salud (OMS) (2019) ^(^ ^18^ ^)^ , que apunta a mapear las ocupaciones de los trabajadores de salud a partir de las diferencias en el nivel de competencias requeridas para la actuación profesional a escala global. Así, fueron considerados cursos de salud: enfermería, farmacia, fisioterapia, fonoaudiología, medicina (alternativa y generalista), *midwifery* , nutrición, odontología y terapia ocupacional. 

 La definición de metaverso siguió el concepto descrito en la revisión de Zhang X, Chen Y, Hu L y Wang Y ^(^ ^3^ ^)^ , que explica esta realidad como un ambiente educativo integrado por tecnologías actualizadas, fundamentales para garantizar los componentes en el mundo real y ficticio con vistas a una experiencia educativa exitosa para los estudiantes. Por tanto, dado el avance científico y la consecuente implementación continua de estrategias digitales en el contexto educativo, surge la necesidad de comprender no sólo la aplicabilidad del metaverso, sino también sus limitaciones para que pueda incorporárselo a la educación de los estudiantes de carreras del área de la salud. 

De esta forma, fue establecida la siguiente pregunta orientadora: “¿Cuál es el estado del conocimiento respecto de la incorporación del metaverso en la educación de estudiantes de carreras del área de la salud?”. Esta pregunta fue dividida en las subpreguntas:

¿Cuál es la aplicabilidad del metaverso en la educación de estudiantes de carreras del área de la salud?

¿Cuáles son las limitaciones del metaverso en la educación de estudiantes de carreras del área de la salud?

### Estrategia de búsqueda

 Fue realizada una búsqueda preliminar en el *Medical Literature Analysis and Retrieval System Online* (MEDLINE) vía PubMed en julio de 2023, con el objetivo de identificar descriptores habituales adoptados por la comunidad científica en relación al tema, considerando que, al momento de las búsquedas en la literatura, “metaverso” no constituía un descriptor. A continuación, se celebraron reuniones entre el equipo de investigación y una bibliotecaria experimentada, a efectos de establecer la estrategia de búsqueda, que fue adecuada y estandarizada en función de las diferentes bases de datos consultadas. 

 Las búsquedas se realizaron el 20 de julio de 2023 en ISI *Web of Science* , MEDLINE a través de PubMed, Embase, Scopus, *EBSCOhost* *Cumulative Index to Nursing and Allied Health* (CINAHL), *BVShost Latin American and Caribbean Health Sciences Literature* (LILACS) y *ProQuest* . Los términos de búsqueda incluyeron: “metaverse*, metaverse environment, metaverse experience, metaverse for learning, metaverse healthcare, metaverse in education, metaverse technolog*”, “educa*, workshop*, training program*, educational activit*, literacy program*, education service, intellectual training, training support” y “health”. Los descriptores y/o palabras clave fueron combinados de acuerdo con los términos del índice *Medical Subject Headings* (MeSH), CINAHL *Titles* , Emtree y Descriptores en Ciencias de la Salud (DeCS) disponibles en cada banco de datos. 

### Criterio de selección

Fueron incluidos registros que mapearan la aplicabilidad y las limitaciones del metaverso en la educación de estudiantes de carreras del área de la salud, publicados en portugués, inglés y/o español (lenguas dominadas por los autores). Fueron excluidos los trabajos estrictamente relacionados a la Realidad Virtual (RV) y Realidad Aumentada (RA) para la educación de estudiantes de carreras del área de la salud. Cabe destacar que no fue adoptado ningún marco temporal.

### Selección de fuentes de evidencias

 Los registros identificados fueron exportados a la *EndNote* ^®^ *Web* para eliminar duplicaciones. Posteriormente, los archivos fueron transferidos a Rayyan ^(^ ^19^ ^)^ para su cribado simultáneo e independiente por parte de dos revisores, a fin de optimizar la confiabilidad y precisión metodológica. Corresponde destacar que se consultó a un tercer revisor experto en el tema a fin de resolver desacuerdos en el proceso de definición de la muestra. El proceso de cribado de los textos completos siguió los mismos procedimientos. A continuación, fue realizada una búsqueda manual y los registros restantes constituyeron la muestra final. 

### Extracción y análisis de datos

Los registros incluidos fueron analizados y sus datos extraídos por un revisor y validados por un segundo, considerando: (1) características del registro: autor, año de publicación y país (original del primer autor); (2) publicación: tipo (discusión o estudio científico), diseño metodológico, población (caracterización y cantidad de participantes, si correspondiere) y principales hallazgos; (3) aplicabilidad del metaverso en la educación de estudiantes de carreras del área de la salud; y (4) limitaciones del metaverso en la educación de estudiantes de carreras del área de la salud. Los datos fueron analizados mediante análisis descriptivo y de contenido, que comprendió información sobre la aplicabilidad y las limitaciones del metaverso en la educación de los estudiantes de carreras del área de la salud.

## Resultados

 Los resultados de este estudio fueron orientados por PRISMA-ScR ^(^ ^16^ ^)^ , cuyo proceso de revisión se describe en la Figura 1 . 

 De los 278 registros mapeados, 23 fueron incluidos en esta revisión ^(^ ^20^ ^-^ ^42^ ^)^ . Dichas producciones fueron publicadas entre 2020 y 2023 en 10 países, distribuidos en cuatro continentes: Asia (11, ~48%) ^(^ ^23^ ^-^ ^26^ ^,^ ^39^ ^,^ ^42^ ^)^ , Europa (5, ~22%) ^(^ ^27^ ^-^ ^29^ ^,^ ^40^ ^-^ ^41^ ^)^ , América (4, ~17%) ^(^ ^30^ ^-^ ^33^ ^)^ y Oceanía (3, ~13%) ^(^ ^20^ ^-^ ^22^ ^)^ . Mayoritariamente, los registros incluidos fueron estudios científicos (17, ~74%) ^(^ ^23^ ^-^ ^29^ ^,^ ^33^ ^-^ ^42^ ^)^ . Entre los métodos utilizados, predominaron los estudios de revisión de la literatura (16, ~70%) ^(^ ^23^ ^,^ ^25^ ^,^ ^27^ ^-^ ^28^ ^,^ ^33^ ^-^ ^42^ ^)^ . Aunque el tipo de revisión no haya sido caracterizado por los autores en su mayoría (10, ~44%) ^(^ ^23^ ^,^ ^25^ ^,^ ^28^ ^,^ ^34^ ^-^ ^38^ ^,^ ^41^ ^-^ ^42^ ^)^ , las revisiones de alcance (2, ~9%) ^(^ ^39^ ^-^ ^40^ ^)^ , las revisiones sistemáticas (1, ~4%) ^(^ ^27^ ^)^ y las revisiones paraguas (1, ~4%) ^(^ ^33^ ^)^ integraron la muestra final. 

 Considerando que esta revisión incluyó varias carreras de salud en un contexto multidisciplinario, se observa que para la mayoría de las producciones (18, ~78%) ^(^ ^20^ ^-^ ^21^ ^,^ ^23^ ^,^ ^25^ ^,^ ^27^ ^-^ ^28^ ^,^ ^30^ ^-^ ^33^ ^,^ ^35^ ^-^ ^42^ ^)^ , la definición de la población por carrera no aplica, considerando que los estudios de revisión de la literatura fueron prevalentes. Sin embargo, un análisis descriptivo muestra que la carrera de medicina es predominante (14, ~61%) ^(^ ^21^ ^,^ ^25^ ^,^ ^28^ ^-^ ^32^ ^,^ ^34^ ^-^ ^36^ ^,^ ^38^ ^-^ ^41^ ^)^ en comparación con enfermería (3, ~13%) ^(^ ^20^ ^,^ ^24^ ^,^ ^33^ ^)^ y odontología (1, ~4%) ^(^ ^42^ ^)^ . La Figura 2 muestra las características de los registros incluidos en esta revisión. 

**Figura 1:** - *Preferred Reporting Items for Systematic Reviews and Meta-Analyses Extension for Scoping Reviews* (PRISMA-ScR) ^(^ ^16^ ^)^ . Ribeirão Preto, SP, Brasil, 2023

**Figura 2:** - Características de los registros incluidos. Ribeirão Preto, SP, Brasil, 2023

**Autores, año de publicación y país**	**Tipo de publicación**	**Diseño metodológico**	**Población**	**Principales hallazgos**
van Weelderen, 2022 ^(^ ^20^ ^)^ Australia	Discusión	Artículo de Opinión	No aplica.	Demuestra que, con el progreso de la tecnología, la educación ha avanzado y, en consecuencia, el papel de la enfermería ha cambiado exponencialmente.
Mah, 2023 ^(^ ^21^ ^)^ Australia	Discusión	Editorial	No aplica.	Destaca que la integración de la Inteligencia Artificial (IA) en la educación, la capacitación y la investigación promete revolucionar la educación y la práctica médicas, ofreciendo mejores resultados a los pacientes.
Moro, 2023 ^(^ ^22^ ^)^ Australia	Discusión	Artículo de Opinión	No detalla.	Demuestra que el metaverso puede ofrecer oportunidades para la enseñanza y el aprendizaje de la anatomía y la fisiología, aunque existen algunos riesgos a considerar.
Chen, Zou, Xie, Wang, 2023 ^(^ ^23^ ^)^ China	Estudio científico	Estudio de Revisión	No aplica.	Demuestra que el metaverso promueve experiencias colaborativas y lúdicas, además del desarrollo de competencias, aunque es necesario resolver algunos desafíos. Las conclusiones destacan la necesidad de medidas de seguridad y de capacitación tecnológica de los instructores, además de explorar el metaverso para monitorear y analizar el comportamiento de los estudiantes.
Yang, Kang, 2022 ^(^ ^24^ ^)^ Corea del Sur	Estudio científico	Cuasiexperimental	58 estudiantes de tercer año de enfermería que concluyeron clases teóricas de esquizofrenia y salud mental relacionada a la comunicación terapéutica, siendo: 29 alumnos para el grupo experimental y 29 alumnos para el grupo control.	Muestra que el metaverso presenta resultados positivos que indican su utilidad como alternativa en la educación en enfermería, superando las limitaciones espaciotemporales de la práctica clínica. Sin embargo, muestra que las limitaciones tecnológicas actuales afectan la naturalidad de las interacciones.
Musamih, Yaqoob, Salah, Jayaraman, Al-Hammadi, Omar, et al., 2023 ^(^ ^25^ ^)^ Emiratos Árabes Unidos	Estudio científico	Estudio de Revisión	No aplica.	Reitera la probable aplicación del metaverso en diversos campos de la salud, como telemedicina, educación médica, marketing de atención médica, cadena de suministros, instalaciones de salud y bienestar. Sin embargo, muestra que la adopción generalizada se enfrenta a importantes desafíos.
Al-Kfairy, Al-Fandi, Alema, Altaee, 2023 ^(^ ^26^ ^)^ Emiratos Árabes Unidos	Estudio científico	Estudio Cualitativo	84 estudiantes de enseñanza superior. Sin embargo, no hubo detalle de las respectivas carreras a las que los estudiantes estaban vinculados.	Destaca que la flexibilidad del aprendizaje es el principal incentivo para utilizar aulas basadas en el metaverso, con otras motivaciones como la preocupación por el medio ambiente y la interactividad que ofrece este tipo de tecnología. Sin embargo, destaca la preocupación por posibles problemas de salud, así como por cuestiones de privacidad y seguridad.
Gonzalez-Moreno, Andrade-Pino, Monfort-Vinuesa, Piñas-Mesa, Rincon, 2023 ^(^ ^27^ ^)^ España	Estudio científico	Revisión Sistemática	No aplica.	Subraya que, para aplicar con éxito estrategias exitosas relacionadas con el metaverso en la educación, es necesario considerar las diversas técnicas de realidad mixta que se han desarrollado, abordando sus potencialidades y fragilidades.
Román-Belmonte, Rodríguez-Merchán, Corte-Rodríguez, 2023 ^(^ ^28^ ^)^ España	Estudio científico	Estudio de Revisión	No aplica.	Señala que el uso del metaverso en la enseñanza de la patología musculoesquelética es promisorio para mejorar la adquisición de conocimientos y promover el aprendizaje independiente. También evidencia las aplicaciones clínicas en desarrollo que podrían agregar valor significativo al campo de la medicina osteoarticular. Asimismo, destaca el uso del metaverso como prometedor, a pesar de cuestiones clínicas, tecnológicas, educativas y legales que aún deben abordarse.
Méndez, Marcos-Pablos, Izard, 2023 ^(^ ^29^ ^)^ España	Estudio Científico	Anales de la 2022 *Tenth International Conference on Technological Ecosystems for* *Enhancing Multiculturality* (TEEM-2022) *Survey*	No detalla.	Señala que el metaverso es eficaz para mejorar la visualización de imágenes reales en ambientes virtuales de aprendizaje. El estudio presenta diversos ambientes virtuales inmersivos y aumentados, utilizando modelos estereoscópicos para crear un metaverso donde poder compartir conocimientos para mejorar la capacitación médica.
López-Ojeda, Hurley, 2023 ^(^ ^30^ ^)^ Estados Unidos de América (EEUU)	Discusión	Comentario	No aplica.	Señala que la tecnología de impresión 3D, sumada a la Inteligencia Artificial (IA), se está desarrollando rápidamente en el contexto del metaverso, impulsando avances en la educación médica y la atención de la salud. Aunque todavía no puede sustituir por completo al uso de cadáveres humanos en la formación médica, el estudio destaca al metaverso como complemento innovador en disciplinas como la neurociencia clínica.
Ford, Buchanan, Azeez, Benrimoh, Kaloiani, Bandeira, et al., 2023 ^(^ ^31^ ^)^ Estados Unidos de América (EEUU)	Discusión	Perspectiva	No aplica.	Destaca que el metaverso ha surgido en el escenario de la educación médica como un ambiente educativo que permite a los estudiantes experimentar situaciones muy cercanas a la realidad, corroborando su razonamiento clínico.
Ahuja, Polascik, Doddapaneni, Byrnes, Sridhar, 2022 ^(^ ^32^ ^)^ Estados Unidos de América (EEUU)	Discusión	Comentario	No aplica.	Señala que, cuando se combina con la Inteligencia Artificial (IA), la Realidad Aumentada (RA) y la Realidad Virtual (RV), el metaverso contribuye a revolucionar la educación médica. También destaca que, aunque aún hay margen para el crecimiento y la consideración de desventajas, su adopción puede ampliar el acceso a la educación médica para las poblaciones desfavorecidas.
De Gagne, Randall, Rushton, Park, Cho, Yamane, et al., 2022 ^(^ ^33^ ^)^ Estados Unidos de América (EEUU)	Estudio científico	Revisión Paraguas	No aplica.	Destaca al metaverso como herramienta pedagógica versátil y en constante evolución, con el potencial de impulsar los resultados del aprendizaje en la educación de enfermería, abriendo oportunidades fascinantes y consideraciones importantes para los docentes.
Bhatia, Joshi, 2023 ^(^ ^34^ ^)^ India	Estudio científico	Anales de la *International Conference on Innovation Data Communication Technologies and Application* 2023 (ICIDCA-2023) Estudio de Revisión	No detalla.	Destaca que la práctica de los estudiantes de medicina en el metaverso está transformando la formación médica, ahorrando tiempo y recursos.
Kawarase, Anjankar, 2022 ^(^ ^35^ ^)^ India	Estudio científico	Estudio de Revisión	No aplica.	Destaca que, al considerar las diferencias culturales en el contexto global, el metaverso puede ofrecer un ambiente educativo que proporciona calidad y uniformidad en la educación médica.
Pandey, Chirputkar, Ashok, 2023 ^(^ ^36^ ^)^ India	Estudio científico	Anales de la *International Conference on Innovative Data Communication Technologies and Application* 2023 (ICIDCA-2023) Estudio de Revisión	No aplica.	Subraya que el metaverso se corresponde con un enfoque educativo que influirá en la educación médica en el futuro, aún estando relacionado con la mejora de determinadas características personales.
Mohamed, Naqishbandi, Veronese, 2023 ^(^ ^37^ ^)^ India	Estudio científico	Estudio de Revisión	No aplica.	Destaca al metaverso como un ambiente nuevo y excepcional para la educación. Evidencia que el tema está en creciente desarrollo, particularmente en lo que se refiere a las aplicaciones, cuya importancia ha aumentado significativamente debido a la pandemia COVID-19.
Bhugaonkar, Bhugaonkar, Masne, 2022 ^(^ ^38^ ^)^ India	Estudio científico	Estudio de Revisión	No aplica.	Señala que el metaverso está intrínsecamente asociado a la Realidad Aumentada (RA), por lo que es posible aplicarlo en el proceso educativo de los estudiantes de medicina.
Garavand, Aslani, 2022 ^(^ ^39^ ^)^ Irán	Estudio científico	Revisión de Alcance	No aplica.	Destaca que, en la educación médica, el uso del metaverso puede revolucionar la forma en que los estudiantes aprenden y practican la medicina, en beneficio de la salud pública.
Petrigna, Musumeci, 2022 ^(^ ^40^ ^)^ Italia	Estudio científico	Revisión de Alcance	No aplica.	Destaca que el metaverso reduce la discrepancia en la formación médica, considerando la eliminación de las barreras geográficas y temporales gracias a las salas virtuales de aprendizaje.
Massetti, Chiariello, 2023 ^(^ ^41^ ^)^ Italia	Estudio científico	Estudio de Revisión	No aplica.	Destaca que, en la educación, el metaverso permite a los estudiantes experimentar situaciones clínicas de forma segura, adquiriendo experiencia en el proceso de enseñanza-aprendizaje.
Bansal, Rajgopal, Chamola, Xiong, Niyato, 2020 ^(^ ^42^ ^)^ Singapur	Estudio científico	Estudio de Revisión	No aplica.	Demuestra que el metaverso surge como alternativa para desarrollar y mejorar los sistemas educativos existentes, permitiendo una experiencia que se asemeja a la realidad.

 La Figura 3 presenta el cuadro-síntesis de los registros incluidos en la muestra final. Los resultados explican la aplicabilidad y las limitaciones del metaverso en la educación de estudiantes de carreras del área de la salud. Aunque algunos estudios detallen solamente uno de los ejes observados, la mayoría (15, ~65%) ^(^ ^21^ ^-^ ^25^ ^,^ ^29^ ^,^ ^31^ ^-^ ^32^ ^,^ ^34^ ^-^ ^35^ ^,^ ^37^ ^-^ ^40^ ^,^ ^42^ ^)^ describe tanto la aplicabilidad como las limitaciones en su totalidad. 

**Figura 3:** - Aplicaciones y limitaciones del metaverso en la educación de estudiantes de carreras del área de la salud. Ribeirão Preto, SP, Brasil, 2023

**Autores y año de publicación**	**Aplicabilidad del metaverso**	**Limitaciones del metaverso**
van Weelderen, 2022 ^(^ ^20^ ^)^	Desarrollo de competencia mediante la ejecución de procedimientos clínicos; Reduce el riesgo de lesiones de los estudiantes al ofrecer un ambiente seguro para practicar y aprender a tomar decisiones críticas antes de interactuar con un paciente real; Reduce el estrés y mejora las tasas de retención del personal.	No describe.
Mah, 2023 ^(^ ^21^ ^)^	Simula escenarios quirúrgicos de la vida real en la educación médica; Permite repetición de prácticas, con recursos ilimitados.	Puede provocar confusión en la comprensión de los estudiantes de la diferenciación entre el metaverso y la vida real. Favorece el fraude académico a través de “trampas” en los exámenes.
Moro, 2023 ^(^ ^22^ ^)^	Disecciones virtuales; Consulta con instructores expertos desde cualquier parte del mundo; Comprensión de la anatomía mediante la representación de órganos en 3D de diferentes modelos anatómicos.	Coste de implementación; Demanda de conectividad estable; Protección, privacidad y seguridad de los datos; Dificultad para monitorear a los estudiantes dentro del mundo virtual.
Chen, Zou, Xie, Wang, 2023 ^(^ ^23^ ^)^	Permite a que los estudiantes amplíen sus competencias clínicas, a partir de situaciones que se asemejan a la vida real.	Dificultades en la seguridad y protección de la privacidad; Puede causar confusión en la comprensión de los alumnos en la asimilación entre lo real y lo virtual.
Yang, Kang, 2022 ^(^ ^24^ ^)^	Permite a los estudiantes mejorar su comunicación terapéutica con los pacientes psiquiátricos.	Las expresiones faciales y las posturas corporales de los avatares son limitadas y distan de ser naturales en comparación con las de los pacientes; Exposición de los programas a problemas que puedan surgir en el proceso operativo durante su uso.
Musamih, Yaqoob, Salah, Jayaraman, Al-Hammadi, Omar, et al., 2023 ^(^ ^25^ ^)^	Facilita la capacitación quirúrgica; Permite la colaboración entre docentes y estudiantes de diferentes localizaciones.	Requiere tecnologías avanzadas y costosas; Requiere recursos relacionados con la interoperabilidad y compatibilidad de los sistemas; Expone al riesgo de ciberdelincuencia (robo de identidad y violación de datos, ciberacoso y hostigamiento); Expone al riesgo de dependencia virtual.
Al-Kfairy, Al-Fandi, Alema, Altaee, 2023 ^(^ ^26^ ^)^	No describe.	Dificultad de acceso y manejo de la tecnología. Problemas de usabilidad ^*^ ; Seguridad y privacidad de los datos.
Gonzalez-Moreno, Andrade-Pino, Monfort-Vinuesa, Piñas-Mesa, Rincon, 2023 ^(^ ^27^ ^)^	Permite la reproducción de *Health Digital Twins* (HDT) ^†^ .	No describe.
Román-Belmonte, Rodríguez-Merchán, Corte-Rodríguez, 2023 ^(^ ^28^ ^)^	Permite la capacitación en habilidades quirúrgicas ortopédicas mediante el reconocimiento de estructuras anatómicas y la ejecución de técnicas fluoroscópicas y suturas quirúrgicas, además de acceso a diversos ambientes quirúrgicos a través de una cámara de 360º; Permite la capacitación de los estudiantes ante situaciones consideradas poco habituales, como las simulaciones con víctimas múltiples; Reduce el riesgo de lesiones accidentales con agujas, bisturís u otros instrumentos cortantes, mejorando la seguridad en los procedimientos invasivos.	No describe.
Méndez, Marcos-Pablos, Izard, 2023 ^(^ ^29^ ^)^	Hace posible la visualización espacial de ambientes reales en el mundo virtual (por ejemplo, quirófanos, salas de disección anatómica, salas de exploración clínica); Hace posible el aprendizaje de las estructuras del cuerpo humano, como el sistema esquelético, la musculatura corporal y otras; Permite a los estudiantes de ciencias de la salud obtener información complementaria al contenido regular de sus estudios, convirtiéndose en participantes activos en el proceso de aprendizaje, estimulándolos durante su progreso educativo.	Aún deben solucionarse aspectos técnicos para hacer real la subjetividad de la sensación del usuario de estar físicamente presente.
López-Ojeda, Hurley, 2023 ^(^ ^30^ ^)^	Respalda la capacitación en procedimientos quirúrgicos en el área de la neurocirugía; Permite la impresión de modelos anatómicos en 3D; Posibilita una presentación mejorada de regiones anatómicas complejas, órganos y estructuras.	No describe.
Ford, Buchanan, Azeez, Benrimoh, Kaloiani, Bandeira, et al., 2023 ^(^ ^31^ ^)^	Promueve *feedback* del desempeño; Promueve la enseñanza especializada (neurosimulación); Promueve la enseñanza simulada (recolección de historia clínica previa; realización de examen físico; generación de hipótesis diagnósticas y proposición de manejo terapéutico de diversas enfermedades, incluyendo las consideradas raras); Promueve la mejora del conocimiento clínico y garantiza la seguridad del paciente a través de oportunidades de aprendizaje.	Implicaciones éticas relacionadas con la accesibilidad y la equidad.
Ahuja, Polascik, Doddapaneni, Byrnes, Sridhar, 2022 ^(^ ^32^ ^)^	Facilita la enseñanza de anatomía y radiología; Ofrece oportunidades para mejorar las habilidades clínicas; Facilita la capacitación quirúrgica.	Altos costos de implementación; Vulnerabilidad en la seguridad de los datos.
De Gagne, Randall, Rushton, Park, Cho, Yamane, et al., 2022 ^(^ ^33^ ^)^	No describe.	Riesgos de mal funcionamiento de la tecnología.
Bhatia, Joshi, 2023 ^(^ ^34^ ^)^	Facilita la capacitación quirúrgica ( manipulación de equipos, habilidades manuales complejas, prácticas en pacientes simulados con datos de pacientes reales); Brinda oportunidades de comunicación e interacción a distancia con otros estudiantes y pacientes; Simulación de casos semejantes a los de la vida real en urgencias neuroquirúrgicas; Ofrece oportunidades para conectar a personas de diferentes regiones.	Alto costo de implementación; Riesgo sobre la privacidad de los datos de los pacientes.
Kawarase, Anjankar, 2022 ^(^ ^35^ ^)^	Posibilita la simulación de escenarios y condiciones de casos que se asemejan a la vida real; Facilita la capacitación en procedimientos quirúrgicos; Posibilita la enseñanza de anatomía de estructuras específicas, como ganglios linfáticos, vasos sanguíneos, nervios, estructuras microscópicas y patologías.	Implementación costosa (infraestructura y personal); Riesgo de violaciones de la seguridad y privacidad de los datos.
Pandey, Chirputkar, Ashok, 2023 ^(^ ^36^ ^)^	Respalda la enseñanza de la anatomía humana; Simula procedimientos quirúrgicos.	No describe.
Mohamed, Naqishbandi, Veronese, 2023 ^(^ ^37^ ^)^	Ofrece un acercamiento entre el mundo real y el virtual.	Tecnologías complejas y no del todo conocidas con potencial para influir profundamente en la vida.
Bhugaonkar, Bhugaonkar, Masne, 2022 ^(^ ^38^ ^)^	Permite la visualización de la estructura anatómica del cuerpo humano; Facilita la capacitación en técnicas quirúrgicas; Posibilita la simulación de procedimientos quirúrgicos de alto costo.	Dificultad o imposibilidad de exportar contenidos entre metaversos; Riesgos relacionados con la privacidad y la protección de los datos de los pacientes; Elevado costo de implementación; Ambiente vulnerable a los ataques cibernéticos.
Garavand, Aslani, 2022 ^(^ ^39^ ^)^	Hizo posible la educación no presencial en el contexto de la pandemia de la COVID-19.	Necesidad de infraestructura para aplicación del metaverso en diferentes áreas.
Petrigna, Musumeci, 2022 ^(^ ^40^ ^)^	Posibilita la capacitación en cirugías en las áreas de cardiología y neurología; Posibilita la visualización de estructuras anatómicas del cuerpo humano.	Fragiliza las conexiones sociales en comparación con la vida real.
Massetti, Chiariello, 2023 ^(^ ^41^ ^)^	Hace posibles clases interactivas de anatomía digital para percibir la evolución del proceso patológico y simular procedimientos; Simula actividades clínicas y de diagnóstico en modelos virtuales utilizando pacientes virtuales con características semejantes a las de los pacientes reales.	No describe.
Bansal, Rajgopal, Chamola, Xiong, Niyato, 2020 ^(^ ^42^ ^)^	Casos simulados con proyecciones en pantalla que permiten hacer un seguimiento de lo que está haciendo el alumno; Enseñanza sobre el posicionamiento de la placenta y las fases del parto; Simuladores odontológicos que responden a la fuerza aplicada por el alumno al simular el procedimiento y le permiten ejercitar los procedimientos y la destreza; Enseñanza sobre atención de urgencias, como reanimación cardiopulmonar, uso de desfibrilador, recolección de muestras para análisis, estudio de anatomía y disección, capacitación quirúrgica.	Cuestiones relacionadas con la privacidad y la seguridad de los datos, leyes y jurisdicción; Riesgos relacionados con la adicción y la salud mental.

^*^ Usabilidad = Se refiere a la facilidad de utilizar una nueva tecnología ^(^ ^26^ ^)^ ;

^†^ *Health Digital Twins* (HDT) = Una representación virtual (gemelo digital) de un paciente (gemelo físico) que se genera a partir de pacientes multimodales ^(^ ^27^ ^)^

## Discusión

Los resultados de este estudio revelan que solo tres de las nueve carreras incluidas en la clasificación de carreras de enseñanza superior en el área de salud retrataron la incorporación del metaverso en la formación de sus estudiantes, escenario en el que la medicina asume el liderazgo. Se observa, por lo tanto, que la incorporación del metaverso a la educación es incipiente y, en consecuencia, son necesarias inversiones que aseguren que la educación en las carreras de pregrado en salud sea equitativa. Las evidencias sintetizadas ponen de manifiesto la aplicabilidad y las limitaciones del metaverso en la enseñanza superior en el área de salud, evidenciando una realidad transversal a la teoría y la práctica.

### Aplicabilidad del metaverso en la educación de estudiantes de carreras del área de la salud

 De hecho, el metaverso respalda la enseñanza a través de la simulación de casos hipotéticos que se asemejan a la realidad ^(^ ^21^ ^,^ ^23^ ^,^ ^27^ ^-^ ^28^ ^,^ ^34^ ^-^ ^35^ ^,^ ^37^ ^,^ ^41^ ^)^ , contribuyendo así al desarrollo de las competencias de los estudiantes. Entre las principales aplicaciones del metaverso, la enseñanza de la anatomía ^(^ ^22^ ^,^ ^28^ ^-^ ^30^ ^,^ ^32^ ^,^ ^35^ ^-^ ^36^ ^,^ ^38^ ^,^ ^40^ ^-^ ^42^ ^)^ fue ampliamente explorada por los registros incluidos, dada su capacidad para facilitar la comprensión de las dimensiones del cuerpo humano a través de la visualización anatómica desde diferentes ángulos. Dicha estrategia constituye un avance respecto de la enseñanza tradicional con cadáveres, particularmente porque permite la observación de estructuras microscópicas ^(^ ^22^ ^,^ ^43^ ^)^ . 

Aún desde una perspectiva teórica, el metaverso contribuye a que el proceso de enseñanza-aprendizaje sea no solo interactivo, sino también atractivo. Por ello, resulta fundamental despertar y estimular la intuición y la imaginación de los estudiantes para que se sientan motivados a aprender, ya que, como ambiente educativo tecnológico, el metaverso tiene mucho que aportar al desempeño académico. Sin embargo, para que ello ocurra, es necesario que los alumnos acepten el metaverso.

 Aunque la aplicabilidad del metaverso esté relacionada con la enseñanza teórica, corrobora el desarrollo de competencias clínicas ^(^ ^20^ ^,^ ^23^ ^-^ ^24^ ^,^ ^28^ ^-^ ^32^ ^,^ ^34^ ^-^ ^35^ ^,^ ^38^ ^,^ ^40^ ^,^ ^42^ ^)^ , tales como la capacitación quirúrgica (independientemente de la especialidad) ^(^ ^25^ ^,^ ^28^ ^-^ ^32^ ^,^ ^34^ ^-^ ^36^ ^,^ ^38^ ^,^ ^40^ ^,^ ^42^ ^)^ y la capacitación ante situaciones de emergencia con múltiples víctimas ^(^ ^31^ ^)^ . Al permitir la repetición de procedimientos ^(^ ^21^ ^)^ sin generar ningún riesgo para pacientes o estudiantes ^(^ ^20^ ^,^ ^28^ ^,^ ^31^ ^)^ , el metaverso hace posible seguir la dinámica del escenario clínico, posibilitando la comprensión de los procedimientos y de la dimensión del equipo de trabajo, e incluso contribuyendo a la enseñanza sobre la seguridad del paciente. 

 Establecida en 2011 por la Organización Mundial de la Salud (OMS), la Guía Curricular Multiprofesional para la Seguridad del Paciente destaca que la seguridad del paciente debe incorporarse a las propuestas pedagógicas de la educación interprofesional en salud ^(^ ^44^ ^)^ . De esta manera, se hace posible la formación de profesionales capaces de actuar de acuerdo con las mejores prácticas, no solo prestando una atención segura y cualificada al paciente, sino también contribuyendo al fortalecimiento de los sistemas de salud. Por ello, se reitera que es esencial invertir en la incorporación de la seguridad del paciente en el proceso de enseñanza-aprendizaje ^(^ ^45^ ^-^ ^47^ ^)^ , constituyendo el metaverso una estrategia posible. 

 En el contexto de la pandemia COVID-19, la inminente necesidad de una educación no presencial hizo propicias, de cierta forma, reflexiones sobre el proceso de enseñanza-aprendizaje ^(^ ^39^ ^)^ . Con la vertiginosa búsqueda de modos de facilitar la interacción y la colaboración entre docentes y estudiantes en distintos lugares ^(^ ^25^ ^)^ , el metaverso recibió un fuerte impulso. Como resultado, las fronteras entre el mundo real y el virtual se han vuelto a veces casi indistinguibles, potenciando la educación y la creación de redes ^(^ ^34^ ^)^ entre estudiantes y docentes de todo el mundo a través de la enseñanza a distancia de emergencia ^(^ ^22^ ^)^ . 

 Es destacable que la síntesis de evidencias contemplara tres de las cuatro posibles aplicaciones del metaverso: aprendizaje combinado, educación basada en competencias y educación inclusiva ^(^ ^3^ ^)^ . En cuanto a la aplicabilidad teórica, el metaverso puede constituir un ambiente educativo tecnológico orientado a promover el protagonismo de los estudiantes en el proceso de enseñanza-aprendizaje. Además, en cuanto a la aplicabilidad práctica, posibilita el desarrollo de competencias clínicas que favorecen la construcción de la identidad profesional, reiterando su compromiso con el sector profesional. 

### Limitaciones del metaverso en la educación de estudiantes de carreras del área de la salud

 Ciertamente, aún queda mucho por avanzar en la incorporación del metaverso a la educación de los estudiantes de carreras del área de la salud. Por lo tanto, debemos alejarnos de la idea sesgada que destaca únicamente la infraestructura como factor necesario para que el metaverso se utilice realmente en la educación. Por otro lado, es fundamental que los docentes cuenten con las competencias necesarias para utilizar este ambiente educativo tecnológico como recurso pedagógico, particularmente considerando la posibilidad de fragilización de las relaciones interpersonales en la díada docente-estudiante. Por esta razón, resulta fundamental que los docentes desarrollen competencias básicas que deben estar impregnadas de conocimientos pedagógicos ^(^ ^48^ ^)^ . 

 A pesar de que la Guía Curricular Multiprofesional para la Seguridad del Paciente no haga mención específica al metaverso, destaca la dificultad de los estudiantes para discernir entre la realidad virtual y la física como una preocupación que debe ser considerada por los docentes ^(^ ^44^ ^)^ . El uso del metaverso por parte de educadores sin conocimientos específicos puede conducir a un proceso de despersonalización de los estudiantes ^(^ ^21^ ^-^ ^23^ ^,^ ^32^ ^,^ ^40^ ^)^ . Como consecuencia, los estudiantes pueden desligarse de la realidad al sentirse desconectados de sus propios cuerpos y pensamientos, ya que el ambiente y las actividades inherentes al metaverso “se parecen” a la vida real (aunque existan limitaciones) ^(^ ^24^ ^,^ ^29^ ^,^ ^33^ ^,^ ^37^ ^)^ , lo cual dificulta la equiparación entre lo real y lo virtual. 

 Sin embargo, en lo que respecta a las limitaciones del metaverso en la educación de los estudiantes de carreras del área de la salud, vale destacar la seguridad y privacidad de los datos ^(^ ^22^ ^-^ ^23^ ^,^ ^25^ ^,^ ^31^ ^-^ ^32^ ^,^ ^34^ ^-^ ^35^ ^,^ ^38^ ^,^ ^42^ ^)^ como factor restrictivo. En la medida en que el mundo real se reproduce en el metaverso, pueden surgir riesgos relacionados con el cibercrimen, como el robo de identidad y la violación de datos, así como el *cyberbullying* y el acoso ^(^ ^25^ ^,^ ^38^ ^)^ . Por esta razón, para la implementación del metaverso es imprescindible considerar los recursos de mantenimiento y protección. En el escenario global, se sabe que la educación superior no es igualitaria, en la medida en que está relacionada con el desarrollo económico ^(^ ^49^ ^)^ . En este sentido, al considerar al metaverso como ambiente tecnológico educativo, los factores limitantes no son sólo su alto costo de implementación ^(^ ^22^ ^,^ ^25^ ^,^ ^32^ ^,^ ^34^ ^-^ ^35^ ^,^ ^38^ ^-^ ^39^ ^)^ , sino también el mantenimiento de la tecnología. 

Se reitera que, a pesar de su aplicabilidad, el metaverso presenta limitaciones que deben considerarse. Para que su adopción sea efectiva, los docentes deben estar capacitados e instruidos en el uso de la tecnología a fin de minimizar las posibles repercusiones negativas para los estudiantes. No sólo para el uso de este ambiente se deben tomar medidas de implementación y estrategias de mantenimiento que garanticen la seguridad y privacidad de los datos. Finalmente, el uso del metaverso puede no ser equitativo, en la medida en que no todas las Instituciones de Educación Superior (IES) cuentan con recursos, condiciones y docentes para implementarlo, lo cual puede contribuir a acentuar la desigualdad en la formación de los estudiantes de carreras del área de la salud.

Este estudio no contempló un análisis por carreras en el área de la salud, ni ahondó en los aspectos físicos y psicológicos relacionados con el uso del metaverso (efectos inducidos por el ambiente virtual: ciberenfermedad, enfermedad del simulador, enfermedad del movimiento, pérdida cognitiva, mareos, malestar físico). Además, al tratarse de un tema emergente en diversas áreas del conocimiento, el concepto de “metaverso” sigue siendo fluido, ya que continuamente se están desarrollando pruebas para calificarlo. Esto puede haber limitado la búsqueda de registros.

## Conclusión

El metaverso contribuye a que el proceso de enseñanza-aprendizaje de los estudiantes de carreras del área de la salud sea no solo interactivo, sino también atractivo. De ese modo, se favorece el desarrollo de competencias clínicas que respaldan la construcción de la identidad profesional. A pesar de ello, el metaverso presenta limitaciones que deben considerarse, particularmente en relación a la seguridad y privacidad de los datos, y al alto costo asociado a su implementación y mantenimiento. El metaverso puede no ser equitativo, en la medida en que no todas las IES cuentan con los recursos, condiciones y profesionales capacitados para implementarlo, lo cual puede contribuir a acentuar la desigualdad en la educación. Las diferentes aplicabilidades del metaverso se han mostrado auspiciosas para la propuesta de una educación superior calificada en el escenario global. De todos modos, tratándose de una ciencia en pleno desarrollo, aún es posible observar desafíos para su incorporación a la educación de estudiantes de carreras del área de la salud.

# Translation: Explorando o metaverso na educação de estudantes da saúde: revisão de escopo ^*^

**DOI:** 10.1590/1518-8345.7256.4348

**Authors:** Andrea Bernardes, Lucas Gardim, Agostinho A C Araújo, Rodrigo Jensen, Raquel Acciarito Motta, Denise Maria de Almeida, Roberta Rubia de Lima, Heloísa Helena Ciqueto Peres

**Affiliations:** ^1^ Universidade de São Paulo, Escola de Enfermagem de Ribeirão Preto, Centro Colaborador da OPAS/OMS para o Desenvolvimento da Pesquisa em Enfermagem, Ribeirão Preto, SP, Brasil.; ^2^ Bolsista do Conselho Nacional de Desenvolvimento Científico e Tecnológico (CNPq), Brasil.; ^3^ Bolsista da University of Alberta Research Award Recipient (NOC 41201), Emerging Leaders in the Americas Program (ELAP) Program, Global Affairs Canada’s International Scholarships Program, Canadá.; ^4^ Universidade de São Paulo, Escola de Enfermagem, São Paulo, SP, Brasil.; ^5^ Prisma Consultoria, São Paulo, SP, Brasil.; ^6^ Secretaria de Saúde do Estado de São Paulo, São Paulo, SP, Brasil.

**Keywords:** Educação, Ensino, Estudantes de Ciências da Saúde, Tecnologia, Tecnologias Educacionais, Realidade Virtual

## Abstract

**Objetivo:**

mapear a literatura a respeito da incorporação do metaverso na educação de estudantes de graduação na área da saúde.

**Método:**

revisão de escopo de acordo com as recomendações do JBI e *Preferred Reporting Items for Systematic Reviews and Meta-Analyses Extension for Scoping Reviews* (PRISMA-ScR), conduzida na *Web of Science* , *Medical Literature Analysis and Retrieval System Online* (MEDLINE) via PubMed, Embase, Scopus, *Cumulative Index to Nursing and Allied Health* (CINAHL), Literatura Latino-Americana e do Caribe em Ciências da Saúde (LILACS) e ProQuest.

**Resultados:**

foram incluídos 23 registros publicados de 2020 a 2023 e desenvolvidos em 10 países. O metaverso destaca-se por permitir a simulação de casos hipotéticos, tornando a educação interativa e atrativa. Apesar disso, enfrenta limitações, incluindo possibilidade de despersonalização dos estudantes, preocupações com segurança de dados e privacidade, além do custo elevado de implementação e manutenção de sua infraestrutura.

**Conclusão:**

o metaverso viabiliza o desenvolvimento de competências clínicas que subsidiam a construção da identidade profissional do estudante. Todavia, pode não ser equitativo, na medida em que demanda recursos e educadores com domínio para implementá-lo, contribuindo para acentuar a desigualdade na educação de estudantes.

## Destaques

**(1)** O metaverso é promissor na educação de estudantes de graduação na área da saúde.

**(2)** O metaverso torna a educação interativa e atrativa.

**(3)** Promove o protagonismo dos estudantes no processo ensino-aprendizagem.

**(4)** Apresenta riscos relacionados à segurança e privacidade de dados.

**(5)** Custo elevado de implementação e manutenção de sua infraestrutura.

## Introdução

 O mundo está em constante evolução tecnológica, que não somente tem influenciado o modo como as pessoas se relacionam como também o processo ensino-aprendizagem, sobretudo na educação de estudantes de graduação na área da saúde ^(^ ^1^ ^-^ ^2^ ^)^ . Nesse campo, tem-se o metaverso como uma das mais recentes e promissoras inovações que oferece uma gama de possibilidades e recursos que transcendem as restrições físicas e geográficas ^(^ ^3^ ^)^ . Por essa razão, tratando-se de um tema de cunho inovador, ainda que os estudantes pertençam a uma geração com maior preparo tecnológico, é imprescindível o entendimento científico a respeito do uso desse ambiente na educação, haja vista as evidências científicas com desfechos positivos relacionados ao uso das tecnologias educativas na melhoria da qualidade da assistência e na qualidade de vida dos pacientes ^(^ ^4^ ^)^ . 

 Tem-se o metaverso como ambiente educacional tecnológico constituído de forma virtual e real, com limites quase indistinguíveis ^(^ ^3^ ^)^ . Embora versado como tema emergente, o termo “metaverso” foi introduzido em 1992 em uma reconhecida obra de ficção científica proposta por Neal Stephenson ^(^ ^5^ ^)^ . Na educação, reitera-se o metaverso como ambiente educacional tecnológico com potencial para revolucionar o processo ensino-aprendizagem, criando ambientes virtuais imersivos e interativos que promovem motivação e engajamento dos estudantes, favorecendo, assim, uma aprendizagem personalizada ^(^ ^6^ ^)^ . 

 Enquanto ambiente tecnológico tridimensional, interativo e imersivo, o metaverso oferece uma gama de possibilidades com as quais as pessoas podem se conectar, colaborar e vivenciar experiências educacionais envolventes ^(^ ^7^ ^-^ ^9^ ^)^ . No contexto de formação do profissional em saúde, evidências mostram a efetividade dessa realidade para a proposição do processo ensino-aprendizagem, na medida em que possibilita a transição da teoria para a prática ^(^ ^10^ ^-^ ^11^ ^)^ . Não obstante, algumas repercussões éticas relativas à aplicação do metaverso são referidas em diversas áreas do conhecimento, além de limitações relacionadas à escassez de recursos para sua implementação e sustentabilidade ^(^ ^8^ ^,^ ^12^ ^)^ . 

 Nesse sentido, ainda que estudos de revisão de literatura tenham sido desenvolvidos evidenciando a aplicação e o uso do metaverso como ambiente educacional ^(^ ^13^ ^-^ ^14^ ^)^ , não sintetizam as evidências no contexto da educação de estudantes de graduação na área da saúde. Tais estudos mostraram que, em virtude de sua capacidade de facilitar interações sociais inovadoras e proporcionar experiências imersivas ^(^ ^13^ ^)^ , o metaverso foi adaptado para o uso no contexto da educação, ao longo do tempo ^(^ ^14^ ^)^ . Por essa razão, este estudo teve como objetivo mapear a literatura a respeito da incorporação do metaverso na educação de estudantes de graduação na área da saúde. 

## Método

 Esta revisão de escopo foi conduzida de acordo com as recomendações do JBI ^(^ ^15^ ^)^ e reportada de acordo com as diretrizes do *Preferred Reporting Items for Systematic Reviews and Meta-Analyses Extension for Scoping Reviews* (PRISMA-ScR) ^(^ ^16^ ^)^ . 

### Protocolo

 Buscas preliminares foram realizadas na literatura e nenhum protocolo ou estudo referente à incorporação do metaverso na educação de estudantes de graduação na área da saúde foi identificado. Em seguida, o protocolo deste estudo foi registrado no *Open Science Framework* (OSF) ^(^ ^17^ ^)^ , de acordo com as recomendações do JBI ^(^ ^15^ ^)^ . 

### Pergunta de pesquisa

 O acrônimo PCC (População, Conceito e Contexto) ^(^ ^15^ ^)^ foi utilizado para estabelecer a questão norteadora, de modo que “P” se refere aos estudantes de graduação, “C” ao metaverso e “C” à educação na área da saúde. 

 Destaca-se que os cursos de educação superior compreendidos na área da saúde foram definidos a partir da classificação proposta pela Organização Mundial da Saúde (OMS) (2019) ^(^ ^18^ ^)^ , que possui como objetivo mapear as ocupações dos trabalhadores de saúde a partir das diferenças assumidas no nível de competências necessárias para a atuação profissional em escala global. Assim, foram considerados como cursos da área da saúde: enfermagem, farmácia, fisioterapia, fonoaudiologia, medicina (alternativa e generalista), *midwifery* , nutrição, odontologia e terapia ocupacional. 

 A definição de metaverso seguiu o conceito descrito na revisão de Zhang X, Chen Y, Hu L e Wang Y ^(^ ^3^ ^)^ , que destaca tal realidade como um ambiente educacional constituído por tecnologias atualizadas, as quais são fundamentais para assegurar os componentes no mundo real e fictício, com vista a uma experiência educacional exitosa aos estudantes. Com isso, dado o progresso científico e consequente implementação contínua de estratégias digitais no contexto da educação, emerge a necessidade de compreender não somente a aplicabilidade do metaverso mas ainda suas limitações para que possa ser incorporado à educação de estudantes de graduação na área da saúde. 

Assim, a seguinte questão norteadora foi estabelecida: “Qual o estado do conhecimento a respeito da incorporação do metaverso na educação de estudantes de graduação na área da saúde?”. Tal questão foi subdividida em subquestões:

Qual a aplicabilidade do metaverso na educação de estudantes de graduação na área da saúde?

Quais são as limitações do metaverso na educação de estudantes de graduação na área da saúde?

### Estratégia de busca

 Uma busca preliminar foi realizada na *Medical Literature Analysis and Retrieval System Online* (MEDLINE) via PubMed, em julho de 2023, com o objetivo de identificar descritores habituais adotados pela comunidade científica relacionados ao tema, considerando que, no momento de realização das buscas na literatura, “metaverso” não se tratava de um descritor. Em seguida, reuniões entre a equipe de pesquisa e uma bibliotecária com experiência foram realizadas para o estabelecimento da estratégia de busca, ajustada e padronizada de acordo com as diferentes bases de dados consultadas. 

 As buscas foram realizadas em 20 de julho de 2023, na ISI *Web of Science* , MEDLINE via PubMed, Embase, Scopus, *EBSCOhost Cumulative Index to Nursing and Allied Health* (CINAHL), *BVShost* Literatura Latino-Americana e do Caribe em Ciências da Saúde (LILACS) e ProQuest. Os termos de busca incluíram: “metaverse*, metaverse environment, metaverse experience, metaverse for learning, metaverse healthcare, metaverse in education, metaverse technolog*” , “educa*, workshop*, training program*, educational activit*, literacy program*, education service, intellectual training, training support” e *“health”* . Descritores e/ou palavras-chave foram combinados de acordo com os termos de índice do *Medical Subject Headings* (MeSH), CINAHL *Titles* , Emtree, e Descritores em Ciências da Saúde (DeCS), disponíveis em cada banco de dados. 

### Critério de seleção

Foram incluídos registros que mapearam a aplicabilidade e as limitações do metaverso na educação de estudantes de graduação na área da saúde publicados em português, inglês e/ou espanhol (línguas de proficiência dos autores). Registros relacionados estritamente à Realidade Virtual (RV) e Realidade Aumentada (RA) para educação de estudantes de graduação na área da saúde foram excluídos. Destaca-se que não foi adotado recorte temporal.

### Seleção de fontes de evidências

 Os registros identificados foram exportados para o *EndNote* ^®^ *Web,* para a remoção de duplicatas. Posteriormente, os arquivos foram transferidos para o Rayyan ^(^ ^19^ ^)^ , para triagem, de maneira simultânea e independente por dois revisores, de modo a aprimorar a confiabilidade e a precisão metodológica. Destaca-se que um terceiro revisor com expertise no tema foi consultado para resolução de discordâncias no processo de definição da amostra. O processo de triagem dos textos completos seguiu os mesmos procedimentos. Em seguida, realizou-se busca manual, e demais registros compuseram a amostra final. 

### Extração e análise de dados

Os registros incluídos foram analisados e tiveram seus dados extraídos por um revisor e validados por um segundo, considerando: (1) características do registro: autor, ano de publicação e país (origem do primeiro autor); (2) publicação: tipo (discussão ou estudo científico), delineamento metodológico, população (caracterização e número de participantes, se aplicável) e principais achados; (3) aplicabilidade do metaverso na educação de estudantes de graduação na área da saúde; e (4) limitações do metaverso na educação de estudantes de graduação na área da saúde. Os dados foram analisados por meio de análise descritiva e de conteúdo que contemplou informações a respeito da aplicabilidade e das limitações do metaverso na educação de estudantes de graduação na área da saúde.

## Resultados

 Os resultados deste estudo foram guiados pelo PRISMA-ScR ^(^ ^16^ ^)^ , cujo processo de revisão foi descrito na Figura 1 . 

 Dos 278 registros mapeados, 23 foram incluídos nesta revisão ^(^ ^20^ ^-^ ^42^ ^)^ . Tais produções foram publicadas entre os anos de 2020 e 2023, em 10 países, distribuídos em quatro continentes: Ásia (11, ~48%) ^(^ ^23^ ^-^ ^26^ ^,^ ^39^ ^,^ ^42^ ^)^ , Europa (5, ~22%) ^(^ ^27^ ^-^ ^29^ ^,^ ^40^ ^-^ ^41^ ^)^ , América (4, ~17%) ^(^ ^30^ ^-^ ^33^ ^)^ e Oceania (3, ~13%) ^(^ ^20^ ^-^ ^22^ ^)^ . Majoritariamente, os registros incluídos corresponderam a estudos científicos (17, ~74%) ^(^ ^23^ ^-^ ^29^ ^,^ ^33^ ^-^ ^42^ ^)^ . Dentre os métodos empregados, foram prevalentes estudos de revisão de literatura (16, ~70%) ^(^ ^23^ ^,^ ^25^ ^,^ ^27^ ^-^ ^28^ ^,^ ^33^ ^-^ ^42^ ^)^ . Ainda que o tipo de revisão não tenha sido caracterizado pelos autores em sua maioria (10, ~44%) ^(^ ^23^ ^,^ ^25^ ^,^ ^28^ ^,^ ^34^ ^-^ ^38^ ^,^ ^41^ ^-^ ^42^ ^)^ , as revisões de escopo (2, ~9%) ^(^ ^39^ ^-^ ^40^ ^)^ , sistemática (1, ~4%) ^(^ ^27^ ^)^ e *umbrella review* (1, ~4%) ^(^ ^33^ ^)^ compuseram a amostra final. 

 Ao considerar que esta revisão integrou diversos cursos da área da saúde em âmbito multidisciplinar, nota-se que, para a maioria das produções (18, ~78%) ^(^ ^20^ ^-^ ^21^ ^,^ ^23^ ^,^ ^25^ ^,^ ^27^ ^-^ ^28^ ^,^ ^30^ ^-^ ^33^ ^,^ ^35^ ^-^ ^42^ ^)^ , a definição da população por curso não se aplica, considerando que foram prevalentes estudos de revisão de literatura. No mais, em uma análise descritiva, observa-se que o curso de medicina é predominante (14, ~61%) ^(^ ^21^ ^,^ ^25^ ^,^ ^28^ ^-^ ^32^ ^,^ ^34^ ^-^ ^36^ ^,^ ^38^ ^-^ ^41^ ^)^ em comparação com a enfermagem (3, ~13%) ^(^ ^20^ ^,^ ^24^ ^,^ ^33^ ^)^ e odontologia (1, ~4%) ^(^ ^42^ ^)^ . A Figura 2 apresenta as características dos registros incluídos nesta revisão. 

**Figura 1:** - *Preferred Reporting Items for Systematic Reviews and Meta-Analyses Extension for Scoping Reviews* ( *PRISMA-ScR* ) ^(^ ^16^ ^)^ . Ribeirão Preto, SP, Brasil, 2023

**Figura 2:** - Características dos registros incluídos. Ribeirão Preto, SP, Brasil, 2023

**Autores, ano de publicação e país**	**Tipo de publicação**	**Delineamento metodológico**	**População**	**Principais achados**
van Weelderen, 2022 ^(^ ^20^ ^)^ Austrália	Discussão	Artigo de Opinião	Não se aplica.	Evidencia que, com o progresso da tecnologia, a educação avançou e, com isso, o papel da enfermagem mudou exponencialmente.
Mah, 2023 ^(^ ^21^ ^)^ Austrália	Discussão	Editorial	Não se aplica.	Destaca que a integração da Inteligência Artificial (IA) na educação, no treinamento e na pesquisa promete revolucionar a educação e prática médica, proporcionando melhores resultados aos pacientes.
Moro, 2023 ^(^ ^22^ ^)^ Austrália	Discussão	Artigo de Opinião	Não detalha.	Demonstra que o metaverso pode oferecer oportunidades para o ensino e aprendizado de anatomia e fisiologia, embora apresente alguns riscos a serem considerados.
Chen, Zou, Xie, Wang, 2023 ^(^ ^23^ ^)^ China	Estudo científico	Estudo de Revisão	Não se aplica.	Evidencia que o metaverso promove experiências colaborativas e lúdicas, além do desenvolvimento de competências, ainda que desafios precisem ser solucionados. As descobertas destacam a necessidade de medidas de segurança e treinamento tecnológico para instrutores, além de explorar o metaverso no monitoramento e análise do comportamento de estudantes.
Yang, Kang, 2022 ^(^ ^24^ ^)^ Coréia do Sul	Estudo científico	Quasi-Experimental	58 estudantes do terceiro ano de enfermagem que concluíram aulas teóricas de esquizofrenia e saúde mental relacionada à comunicação terapêutica, sendo: 29 alunos para o grupo experimental e 29 alunos para o grupo controle.	Evidencia que o metaverso apresenta resultados positivos que indicam sua utilidade como alternativa na educação em enfermagem, superando limitações espaço-temporais da prática clínica. No entanto, evidencia que as limitações tecnológicas atuais afetam a naturalidade das interações.
Musamih, Yaqoob, Salah, Jayaraman, Al-Hammadi, Omar, et al., 2023 ^(^ ^25^ ^)^ Emirados Árabes Unidos	Estudo científico	Estudo de Revisão	Não se aplica.	Reitera a provável aplicação do metaverso em diversos campos da saúde, como telemedicina, educação médica, *marketing* de assistência médica, cadeia de suprimentos, instalações de saúde e bem-estar. No entanto, mostra que a adoção generalizada enfrenta desafios significativos.
Al-Kfairy, Al-Fandi, Alema, Altaee, 2023 ^(^ ^26^ ^)^ Emirados Árabes Unidos	Estudo científico	Estudo Qualitativo	84 estudantes do ensino superior. Entretanto, não houve detalhamento dos respectivos cursos aos quais os estudantes estavam vinculados.	Ressalta que a flexibilidade de aprendizado é o principal incentivo para o uso de salas de aula baseadas no metaverso, com outras motivações incluindo a preocupação com o meio ambiente e a interatividade oferecida por essa forma de tecnologia. No entanto, preocupações com possíveis problemas de saúde são evidenciadas, além de questões de privacidade e segurança.
Gonzalez-Moreno, Andrade-Pino, Monfort-Vinuesa, Piñas-Mesa, Rincon, 2023 ^(^ ^27^ ^)^ Espanha	Estudo científico	Revisão Sistemática	Não se aplica.	Enfatiza que, para implementar estratégias bem-sucedidas relacionadas ao metaverso na educação, necessita-se considerar as diversas técnicas de realidade mista desenvolvidas, abordando suas potencialidades e fragilidades.
Román-Belmonte, Rodríguez-Merchán, Corte-Rodríguez, 2023 ^(^ ^28^ ^)^ Espanha	Estudo científico	Estudo de Revisão	Não se aplica.	Aponta o uso do metaverso na educação em patologia músculo-esquelética como promissor para aprimorar a aquisição de conhecimento e promover a aprendizagem independente. Além disso, evidencia aplicações clínicas em desenvolvimento que podem agregar valor significativo ao campo da medicina osteoarticular. No mais, destaca o uso do metaverso como promissor, apesar de questões clínicas, tecnológicas, educacionais e legais que ainda precisam ser endereçadas.
Méndez, Marcos-Pablos, Izard, 2023 ^(^ ^29^ ^)^ Espanha	Estudo Científico	Anais do 2022 *Tenth International Conference on Technological Ecosystems for* *Enhancing Multiculturality* (TEEM-2022) *Survey*	Não detalha.	Ressalta que o metaverso é eficiente no aprimoramento da visualização de imagens reais em ambientes virtuais de aprendizagem. O estudo apresenta diversos ambientes virtuais imersivos e aumentados, utilizando modelos de estereoscopia para criar um metaverso onde o conhecimento pode ser compartilhado para melhorar o treinamento médico.
López-Ojeda, Hurley, 2023 ^(^ ^30^ ^)^ Estados Unidos da América (EUA)	Discussão	Comentário	Não se aplica.	Aponta que a tecnologia de impressão tridimensional, aliada à Inteligência Artificial (IA), está se desenvolvendo rapidamente no contexto do metaverso, impulsionando avanços na educação médica e nos cuidados de saúde. Embora ainda não possa substituir completamente o uso de cadáveres humanos no treinamento médico, o estudo evidencia o metaverso como complemento inovador para disciplinas como neurociência clínica.
Ford, Buchanan, Azeez, Benrimoh, Kaloiani, Bandeira, et al., 2023 ^(^ ^31^ ^)^ Estados Unidos da América (EUA)	Discussão	Perspectiva	Não se aplica.	Enfatiza que o metaverso emergiu no cenário da educação médica como ambiente educacional que propicia a vivência de situações que beiram a realidade, corroborando o raciocínio clínico de estudantes.
Ahuja, Polascik, Doddapaneni, Byrnes, Sridhar, 2022 ^(^ ^32^ ^)^ Estados Unidos da América (EUA)	Discussão	Comentário	Não se aplica.	Aponta que, quando combinado à Inteligência Artificial (IA), Realidade Aumentada (RA) e Realidade Virtual (VR), o metaverso contribui para revolucionar a educação médica. Ainda, destaca que, embora ainda haja espaço para crescimento e consideração de desvantagens, sua adoção pode ampliar o acesso à educação médica para populações carentes.
De Gagne, Randall, Rushton, Park, Cho, Yamane, et al., 2022 ^(^ ^33^ ^)^ Estados Unidos da América (EUA)	Estudo científico	*Umbrella Review*	Não se aplica.	Ressalta o metaverso como ferramenta pedagógica versátil e em constante evolução, com o potencial para impulsionar os resultados de aprendizagem na educação em enfermagem, abrindo oportunidades fascinantes e considerações importantes para educadores.
Bhatia, Joshi, 2023 ^(^ ^34^ ^)^ Índia	Estudo científico	Anais do *International Conference on Innovation Data Communication Technologies and Application* 2023 (ICIDCA-2023) Estudo de Revisão	Não detalha.	Destaca que a prática dos estudantes de medicina no metaverso está transformando o treinamento médico, economizando tempo e recursos.
Kawarase, Anjankar, 2022 ^(^ ^35^ ^)^ Índia	Estudo científico	Estudo de Revisão	Não se aplica.	Destaca que, ao considerar as diferenças culturais no contexto global, o metaverso pode oferecer um ambiente educacional que proporciona qualidade e uniformidade na educação médica.
Pandey, Chirputkar, Ashok, 2023 ^(^ ^36^ ^)^ Índia	Estudo científico	Anais do *International Conference on Innovative Data Communication Technologies and Application* 2023 (ICIDCA-2023) Estudo de Revisão	Não se aplica.	Ressalta que o metaverso corresponde a uma abordagem educacional que influenciará a educação médica no futuro, ainda que esteja relacionada ao aprimoramento de algumas características pessoais.
Mohamed, Naqishbandi, Veronese, 2023 ^(^ ^37^ ^)^ Índia	Estudo científico	Estudo de Revisão	Não se aplica.	Destaca o metaverso como um ambiente novo e excepcional para a educação. Evidencia que o tema está em crescente desenvolvimento, especialmente no que diz respeito às aplicações, cuja importância aumentou significativamente em razão da pandemia COVID-19.
Bhugaonkar, Bhugaonkar, Masne, 2022 ^(^ ^38^ ^)^ Índia	Estudo científico	Estudo de Revisão	Não se aplica.	Aponta que o metaverso está intrinsecamente associado à Realidade Aumentada (RA), sendo, portanto, possível aplicá-lo no processo educativo de estudantes de medicina.
Garavand, Aslani, 2022 ^(^ ^39^ ^)^ Irã	Estudo científico	Revisão de Escopo	Não se aplica.	Destaca que, na educação médica, o uso do metaverso pode revolucionar a forma como os estudantes aprendem e praticam a medicina, beneficiando a saúde pública.
Petrigna, Musumeci, 2022 ^(^ ^40^ ^)^ Itália	Estudo científico	Revisão de Escopo	Não se aplica.	Enfatiza que o metaverso reduz a discrepância na formação médica, considerando a eliminação de barreiras geográficas e temporais por meio de salas virtuais de aprendizagem.
Massetti, Chiariello, 2023 ^(^ ^41^ ^)^ Itália	Estudo científico	Estudo de Revisão	Não se aplica.	Ressalta que, na educação, o metaverso possibilita que os estudantes vivenciem situações clínicas com segurança, adquirindo experiência no processo ensino-aprendizagem.
Bansal, Rajgopal, Chamola, Xiong, Niyato, 2020 ^(^ ^42^ ^)^ Singapura	Estudo científico	Estudo de Revisão	Não se aplica.	Demonstra que o metaverso surge como alternativa para desenvolver e melhorar os sistemas educacionais existentes, o que possibilita uma experiência que se assemelha à realidade.

 A Figura 3 apresenta o quadro-síntese dos registros incluídos na amostra final. Os resultados elucidam a aplicabilidade e as limitações do metaverso na educação de estudantes de graduação da área da saúde. Ainda que alguns estudos detalhem somente um dos eixos observados, a maioria (15, ~65%) ^(^ ^21^ ^-^ ^25^ ^,^ ^29^ ^,^ ^31^ ^-^ ^32^ ^,^ ^34^ ^-^ ^35^ ^,^ ^37^ ^-^ ^40^ ^,^ ^42^ ^)^ descreve tanto a aplicabilidade quanto as limitações em sua totalidade. 

**Figura 3:** - Aplicações e limitações do metaverso na educação de estudantes de graduação da área da saúde. Ribeirão Preto, SP, Brasil, 2023

**Autores e ano de publicação**	**Aplicabilidade do metaverso**	**Limitações do metaverso**
van Weelderen, 2022 ^(^ ^20^ ^)^	Desenvolvimento de competências por meio da execução de procedimentos clínicos; Reduz o risco de lesões aos estudantes, na medida em que oferece um ambiente seguro para prática e aprendizado na tomada de decisões críticas, antes de interagir com um paciente real; Reduz o estresse e melhora as taxas de retenção de pessoal.	Não descreve.
Mah, 2023 ^(^ ^21^ ^)^	Simula cenários cirúrgicos da vida real na educação médica; Permite práticas repetidas, com recursos ilimitados.	Pode causar confundimento na compreensão dos estudantes na diferenciação entre o metaverso e a vida real; Favorece fraudes acadêmicas por meio de ’’cola” em provas.
Moro, 2023 ^(^ ^22^ ^)^	Dissecações virtuais; Consulta com instrutores especialistas de qualquer lugar do mundo; Compreensão da anatomia por meio da representação de órgãos 3D de diferentes modelos anatômicos.	Custo de implementação; Demanda de conectividade estável; Proteção, privacidade e segurança dos dados; Dificuldade para monitoramento dos alunos inseridos no mundo virtual.
Chen, Zou, Xie, Wang, 2023 ^(^ ^23^ ^)^	Possibilita a expansão das competências clínicas dos estudantes, pautada em situações que se assemelham às da vida real.	Dificuldades na segurança e proteção de privacidade; Pode causar confundimento na compreensão dos estudantes na assimilação entre o real e o virtual.
Yang, Kang, 2022 ^(^ ^24^ ^)^	Viabiliza o aprimoramento da comunicação terapêutica de estudantes com pacientes psiquiátricos.	Expressões faciais e posturas corporais dos avatares são limitadas e longe de serem naturais em comparação com as dos pacientes; Exposição dos programas a problemas que podem ocorrer no processo operacional durante o uso.
Musamih, Yaqoob, Salah, Jayaraman, Al-Hammadi, Omar, et al., 2023 ^(^ ^25^ ^)^	Viabiliza o treinamento cirúrgico; Facilita a colaboração entre educadores e estudantes de diferentes localidades.	Demanda tecnologias avançadas e caras; compatibi Demanda recursos relativos à interoperabilidade e lidade dos sistemas; Expõe ao risco de cibercrimes (roubo de identidade e violação de dados, *cyberbullying* e assédio); Expõe ao risco de dependência virtual.
Al-Kfairy, Al-Fandi, Alema, Altaee, 2023 ^(^ ^26^ ^)^	Não descreve.	Dificuldade no acesso e manejo da tecnologia; Problemas de usabilidade ^*^ ; Segurança e privacidade dos dados.
Gonzalez-Moreno, Andrade-Pino, Monfort-Vinuesa, Piñas-Mesa, Rincon, 2023 ^(^ ^27^ ^)^	Possibilita a reprodução de *Health Digital Twins (HDT)* ^†^ .	Não descreve.
Román-Belmonte, Rodríguez-Merchán, Corte-Rodríguez, 2023 ^(^ ^28^ ^)^	Oportuniza o treinamento de habilidades cirúrgicas ortopédicas pelo reconhecimento de estruturas anatômicas e a realização de técnicas fluoroscópicas e suturas cirúrgicas, além do acesso a diversos ambientes cirúrgicos através de câmera 360º; Oportuniza o treinamento dos estudantes em situações consideradas raras, como simulações de múltiplas vítimas; Reduz o risco de lesões acidentais com agulhas, bisturis ou outros instrumentos cortantes, melhorando a segurança em procedimentos invasivos.	Não descreve.
Méndez, Marcos-Pablos, Izard, 2023 ^(^ ^29^ ^)^	Possibilita a visualização espacial de ambientes reais no mundo virtual (por exemplo, salas de operação, salas de dissecção anatômica, exame clínico/salas); Possibilita o aprendizado sobre as estruturas do corpo humano, como o sistema esquelético, a musculatura corporal e outros; Permite que estudantes de ciências da saúde obtenham informações complementares ao conteúdo regular de seus estudos, tornando-os participantes ativos do processo de aprendizagem, incentivando-os durante seu progresso educacional.	Aspectos técnicos ainda precisam ser solucionados, para tornar real a subjetividade da sensação do usuário de estar fisicamente presente.
López-Ojeda, Hurley, 2023 ^(^ ^30^ ^)^	Corrobora o treinamento de procedimentos cirúrgicos na área de neurocirurgia; Viabiliza a impressão de modelos anatômicos 3D; Possibilita a apresentação aprimorada de regiões anatômicas complexas, órgãos e estruturas.	Não descreve.
Ford, Buchanan, Azeez, Benrimoh, Kaloiani, Bandeira, et al., 2023 ^(^ ^31^ ^)^	Promove *feedback* de desempenho; Promove o ensino especializado (neuro-simulação); Promove o ensino simulado (coleta de histórico prévio; realização de exame físico; geração de hipóteses diagnósticas e proposição de manejo terapêutico de diversas doenças, inclusive as consideradas raras); Promove o aprimoramento do conhecimento clínico e garante a segurança do paciente por meio de oportunidades de aprendizagem.	Implicações éticas relacionadas à acessibilidade e equidade.
Ahuja, Polascik, Doddapaneni, Byrnes, Sridhar, 2022 ^(^ ^32^ ^)^	Viabiliza o ensino de anatomia e radiologia; Oportuniza o aprimoramento de competências clínicas; Viabiliza o treinamento cirúrgico.	Alto custo de implementação; Vulnerabilidade na segurança de dados.
De Gagne, Randall, Rushton, Park, Cho, Yamane, et al., 2022 ^(^ ^33^ ^)^	Não descreve.	Riscos de mau funcionamento da tecnologia.
Bhatia, Joshi, 2023 ^(^ ^34^ ^)^	Viabiliza o treinamento cirúrgico (manuseio de equipamentos, habilidades manuais complexas, prática em pacientes simulados com dados de pacientes reais); Oportuniza a comunicação e interação remota com outros estudantes e pacientes; Simulação de casos que se assemelham aos da vida real na área de emergência neurocirúrgica; Oportuniza a conexão de pessoas de diversas regiões.	Alto custo para a implementação; Risco acerca da privacidade dos dados dos pacientes.
Kawarase, Anjankar, 2022 ^(^ ^35^ ^)^	Possibilita a simulação de cenários e condições de casos que se assemelham aos da vida real; Viabiliza o treinamento de procedimentos cirúrgicos; Possibilita o ensino de anatomia de estruturas específicas, como gânglios linfáticos, vasos sanguíneos, nervos, estruturas microscópicas e patologias.	Demanda custo de implementação (infraestrutura e pessoal); Risco de violação de segurança e privacidade de dados.
Pandey, Chirputkar, Ashok, 2023 ^(^ ^36^ ^)^	Corrobora o ensino da anatomia humana; Simula procedimentos cirúrgicos.	Não descreve.
Mohamed, Naqishbandi, Veronese, 2023 ^(^ ^37^ ^)^	Proporciona uma assimilação entre o mundo real e o virtual.	Tecnologias complexas e não totalmente compreendidas, com potencial para influenciar profundamente a vida.
Bhugaonkar, Bhugaonkar, Masne, 2022 ^(^ ^38^ ^)^	Possibilita a visualização da estrutura anatômica do corpo humano; Viabiliza o treinamento de técnicas cirúrgicas; Possibilita a simulação de procedimentos cirúrgicos que possuem alto custo.	Dificuldade ou impossibilidade de exportação de conteúdos entre metaversos; Riscos relacionados à privacidade e proteção dos dados dos pacientes; Alto custo de implementação; Ambiente vulnerável aos ataques cibernéticos.
Garavand, Aslani, 2022 ^(^ ^39^ ^)^	Oportunizou a educação não presencial no contexto pandêmico da COVID-19.	Necessidade de infraestrutura para aplicação do metaverso em diferentes áreas.
Petrigna, Musumeci, 2022 ^(^ ^40^ ^)^	Viabiliza o treinamento em cirurgias nas áreas de cardiologia e neurologia; Possibilita a visualização de estruturas anatômicas do corpo humano.	Fragiliza as conexões sociais em comparação com a vida real.
Massetti, Chiariello, 2023 ^(^ ^41^ ^)^	Viabiliza aulas de anatomia digitais interativas, de modo a perceber a evolução do processo patológico e simular procedimentos; Simula atividades clínicas e diagnósticas em modelos virtuais, por meio de pacientes virtuais com características semelhantes às dos pacientes reais.	Não descreve.
Bansal, Rajgopal, Chamola, Xiong, Niyato, 2020 ^(^ ^42^ ^)^	Casos simulados com projeção em tela que permitem que se possa acompanhar o que está sendo realizado pelo estudante; Ensino sobre o posicionamento da placenta e as fases do parto; Simuladores odontológicos que respondem à força aplicada pelo estudante ao simular o procedimento e permitem treinar procedimentos e destreza; Ensino sobre cuidados de emergência, como ressuscitação cardiopulmonar, uso de desfibrilador, coleta de exames, estudo de anatomia e dissecação, e treinamento cirúrgico.	Questões relativas à privacidade e segurança dos dados, leis e jurisdição; Riscos relacionados ao vício e saúde mental.

^*^ Usabilidade = Refere-se à facilidade de utilizar uma nova tecnologia ^(^ ^26^ ^)^ ;

^†^ *Health Digital Twins (HDT)* = Uma representação virtual (gêmeo digital) de um paciente (gêmeo físico) que é gerada a partir de pacientes multimodais ^(^ ^27^ ^)^

## Discussão

Os resultados deste estudo revelam que apenas três dos nove cursos compreendidos na classificação dos cursos de educação superior da área da saúde retrataram a incorporação do metaverso na educação de estudantes, cenário em que a medicina assume protagonismo. Constata-se, portanto, que a incorporação do metaverso na educação é incipiente e, com isso, investimentos são necessários para que a educação nos cursos de graduação da área da saúde seja equitativa. As evidências sintetizadas destacam aplicabilidade e limitações do metaverso no ensino superior na área da saúde, evidenciando uma realidade que perpassa a teoria e a prática.

### Aplicabilidade do metaverso na educação de estudantes da graduação na área da saúde

 De fato, o metaverso corrobora a educação por meio da simulação de casos hipotéticos que se assemelham à realidade ^(^ ^21^ ^,^ ^23^ ^,^ ^27^ ^-^ ^28^ ^,^ ^34^ ^-^ ^35^ ^,^ ^37^ ^,^ ^41^ ^)^ , contribuindo, com isso, para o desenvolvimento de competências dos estudantes. Dentre as principais aplicabilidades do metaverso, o ensino de anatomia ^(^ ^22^ ^,^ ^28^ ^-^ ^30^ ^,^ ^32^ ^,^ ^35^ ^-^ ^36^ ^,^ ^38^ ^,^ ^40^ ^-^ ^42^ ^)^ foi, em grande parte, explorado pelos registros incluídos, dada sua capacidade de facilitar a compreensão das dimensões do corpo humano por meio da visualização anatômica em diferentes ângulos. Tal estratégia corresponde a um avanço no ensino tradicional com cadáveres, sobretudo ao possibilitar a observação de estruturas microscópicas ^(^ ^22^ ^,^ ^43^ ^)^ . 

Ainda em uma perspectiva teórica, tem-se que o metaverso contribui para que o processo ensino-aprendizagem seja não apenas interativo mas também atrativo. Assim, é fundamental que a intuição e a imaginação dos estudantes sejam despertadas e estimuladas para que possam estar motivados ao aprendizado, na medida em que, enquanto ambiente educacional tecnológico, o metaverso muito tem a contribuir para o desempenho acadêmico. Entretanto, para que isso ocorra, faz-se necessária a aceitação do metaverso pelos estudantes.

 Ainda que a aplicabilidade do metaverso esteja relacionada ao ensino teórico, corrobora o desenvolvimento de competências clínicas ^(^ ^20^ ^,^ ^23^ ^-^ ^24^ ^,^ ^28^ ^-^ ^32^ ^,^ ^34^ ^-^ ^35^ ^,^ ^38^ ^,^ ^40^ ^,^ ^42^ ^)^ , a exemplo do treinamento cirúrgico (independentemente da especialidade) ^(^ ^25^ ^,^ ^28^ ^-^ ^32^ ^,^ ^34^ ^-^ ^36^ ^,^ ^38^ ^,^ ^40^ ^,^ ^42^ ^)^ e do treinamento de situações de urgência com múltiplas vítimas ^(^ ^31^ ^)^ . Ao permitir a repetição de procedimentos ^(^ ^21^ ^)^ sem ocasionar riscos ao paciente e ao estudante ^(^ ^20^ ^,^ ^28^ ^,^ ^31^ ^)^ , é possível, por meio do metaverso, acompanhar a dinâmica do cenário clínico, oportunizando a compreensão dos procedimentos e da dimensão da equipe de trabalho e contribuindo, até mesmo, para o ensino da segurança do paciente. 

 Estabelecido em 2011 pela Organização Mundial da Saúde (OMS), o *Multi-Professional Patient Safety Curriculum Guide* ressalta que a segurança do paciente deve ser incorporada às propostas pedagógicas da educação interprofissional em saúde ^(^ ^44^ ^)^ . Desse modo, torna-se possível a formação de profissionais aptos para atuar em conformidade com as melhores práticas, não somente oportunizando o oferecimento de um cuidado seguro e qualificado ao paciente mas ainda contribuindo para o fortalecimento dos sistemas de saúde. À vista disso, reitera-se a imprescindibilidade do investimento para a incorporação da segurança do paciente no processo ensino-aprendizagem ^(^ ^45^ ^-^ ^47^ ^)^ , sendo o metaverso uma possível estratégia. 

 No contexto da pandemia COVID-19, a necessidade iminente da educação não presencial subvencionou, de certa forma, reflexões sobre o processo ensino-aprendizagem ^(^ ^39^ ^)^ . Com a vertiginosa busca por meios para facilitar a interação e colaboração entre educadores e estudantes em diferentes localidades ^(^ ^25^ ^)^ , o metaverso foi impulsionado. Com isso, os limites entre o universo real e virtual se tornaram, por vezes, quase indistinguíveis, potencializando a educação e o *networking* ^(^ ^34^ ^)^ entre estudantes e educadores ao redor do mundo, por meio do ensino remoto emergencial ^(^ ^22^ ^)^ . 

 É notório que a síntese das evidências contemplou três de quatro aplicabilidades possíveis do metaverso, sendo elas: aprendizagem combinada, educação baseada em competências e educação inclusiva ^(^ ^3^ ^)^ . No que diz respeito à aplicabilidade teórica, o metaverso pode se constituir como ambiente educacional tecnológico direcionado à promoção do protagonismo dos estudantes no processo ensino-aprendizagem. Para além disso, com relação à aplicabilidade prática, viabiliza o desenvolvimento de competências clínicas que subsidiam a construção da identidade profissional, reiterando o seu compromisso com o profissionalismo. 

### Limitações do metaverso na educação de estudantes da graduação na área da saúde

 Certamente, ainda há muito a se avançar para a incorporação do metaverso na educação de estudantes de graduação na área da saúde. Desvencilha-se, portanto, da ideia que ressalta apenas a infraestrutura como fator necessário para que o metaverso possa de fato ser empreendido na educação. Antagonicamente, é fundamental que os educadores possuam domínio para utilizar esse ambiente educacional tecnológico como recurso pedagógico, especialmente ao considerar a possibilidade de fragilização do relacionamento interpessoal no que concerne à díade educador-estudante. Por isso, é indispensável o desenvolvimento de competências básicas pelos educadores que devem estar imbuídos de conhecimentos pedagógicos ^(^ ^48^ ^)^ . 

 Embora o *Multi-Professional Patient Safety Curriculum Guide* não mencione especificamente a respeito do metaverso, tal documento ressalta a dificuldade em discernir a realidade virtual da física por parte dos estudantes como uma preocupação que deve ser considerada pelos educadores ^(^ ^44^ ^)^ . O uso do metaverso por educadores que não apresentem o conhecimento específico pode culminar em um processo de despersonalização dos estudantes ^(^ ^21^ ^-^ ^23^ ^,^ ^32^ ^,^ ^40^ ^)^ . Com isso, os estudantes podem ser afastados da realidade ao se sentirem desconectados de seu próprio corpo e seus pensamentos, visto que o ambiente e as atividades inerentes ao metaverso se “assemelham” aos da vida real (ainda que ele apresente limitações) ^(^ ^24^ ^,^ ^29^ ^,^ ^33^ ^,^ ^37^ ^)^ , tornando difícil equiparar o que é real ao que é virtual. 

 Não obstante, tratando-se das limitações do metaverso na formação de estudantes de graduação na área da saúde, vale ressaltar a segurança e a privacidade dos dados ^(^ ^22^ ^-^ ^23^ ^,^ ^25^ ^,^ ^31^ ^-^ ^32^ ^,^ ^34^ ^-^ ^35^ ^,^ ^38^ ^,^ ^42^ ^)^ como fator restritivo. Na medida em que o mundo real é reproduzido no metaverso, riscos relativos a cibercrimes, como o roubo de identidade e violação dos dados, bem como *cyberbullying* e assédio ^(^ ^25^ ^,^ ^38^ ^)^ , podem ser acarretados. Por essa razão, para que a implementação do metaverso ocorra, é imprescindível que recursos de manutenção e proteção sejam ponderados. No cenário global, sabe-se que a educação superior não é igualitária, na medida em que está relacionada com o desenvolvimento econômico ^(^ ^49^ ^)^ . Nesse sentido, ao considerar o metaverso como ambiente educacional tecnológico, destacam-se como fatores limitantes não somente o seu alto custo de implementação ^(^ ^22^ ^,^ ^25^ ^,^ ^32^ ^,^ ^34^ ^-^ ^35^ ^,^ ^38^ ^-^ ^39^ ^)^ mas também a manutenção da tecnologia. 

Reitera-se que, apesar de sua aplicabilidade, o metaverso apresenta limitações que devem ser consideradas. Para que sua adoção seja efetiva, os educadores devem ser habilitados e instruídos para o uso da tecnologia, a fim de minimizar possíveis repercussões negativas aos estudantes. Não somente para o uso desse ambiente, medidas de implementação devem ser tomadas juntamente com estratégias de manutenção, para que a segurança e privacidade dos dados possam ser garantidas. Por fim, o uso do metaverso pode não ser equitativo, na medida em que nem todas as Instituições de Ensino Superior (IES) apresentam recursos, condições e educadores com domínio para implementá-lo, o que pode contribuir para acentuar a desigualdade da educação de estudantes de graduação na área da saúde.

Este estudo não contemplou análise por cursos compreendidos na área da saúde bem como aspectos físicos e psicológicos relacionados ao uso do metaverso (efeitos induzidos pelo ambiente virtual: doença cibernética, doença do simulador, doença do movimento, perda cognitiva, tonturas, desconforto físico). Além disso, tratando-se de um tema emergente em diversas áreas do conhecimento, o conceito de ‘’metaverso’’ é ainda fluido, na medida em que evidências estão continuamente sendo desenvolvidas a fim de qualificá-lo. Isso pode ter limitado a busca dos registros.

## Conclusão

O metaverso contribui para que o processo ensino-aprendizagem de estudantes de graduação na área da saúde seja não apenas interativo mas também atrativo. Com isso, viabiliza-se o desenvolvimento de competências clínicas que subsidiam a construção da identidade profissional. Apesar disso, o metaverso apresenta limitações que devem ser consideradas, sobretudo com relação à segurança e privacidade dos dados e ao alto custo associado à sua implementação e manutenção. O metaverso pode não ser equitativo, visto que nem todas as IES apresentam recursos, condições e profissionais com domínio para implementá-lo, o que pode contribuir para acentuar a desigualdade na educação. As diferentes aplicabilidades do metaverso têm se mostrado auspiciosas para proposição de uma educação superior qualificada no cenário global. Todavia, enquanto ciência em desenvolvimento, desafios ainda são observados para a sua incorporação na educação de estudantes de graduação na área da saúde.
